# Single‐cell transcriptome dissecting the microenvironment remodeled by PD1 blockade combined with photodynamic therapy in a mouse model of oral carcinogenesis

**DOI:** 10.1002/mco2.636

**Published:** 2024-07-02

**Authors:** Yunmei Dong, Kan Zeng, Ruixue Ai, Chengli Zhang, Fei Mao, Hongxia Dan, Xin Zeng, Ning Ji, Jing Li, Xin Jin, Qianming Chen, Yu Zhou, Taiwen Li

**Affiliations:** ^1^ State Key Laboratory of Oral Diseases National Clinical Research Center for Oral Diseases Chinese Academy of Medical Sciences Research Unit of Oral Carcinogenesis and Management West China Hospital of Stomatology Sichuan University Chengdu China; ^2^ Chongqing Key Laboratory of Oral Diseases, College of Stomatology, Chongqing Medical University Chongqing China; ^3^ State Institute of Drug/Medical Device Clinical Trial West China Hospital of Stomatology Chengdu China; ^4^ Collaborative Innovation Center for Cancer Personalized Medicine Nanjing Medical University Nanjing China

**Keywords:** immune checkpoint blockade, multiomics, oral carcinogenesis, photodynamic therapy, single‐cell transcriptome sequencing

## Abstract

Oral squamous cell carcinoma (OSCC) stands as a predominant and perilous malignant neoplasm globally, with the majority of cases originating from oral potential malignant disorders (OPMDs). Despite this, effective strategies to impede the progression of OPMDs to OSCC remain elusive. In this study, we established mouse models of oral carcinogenesis via 4‐nitroquinoline 1‐oxide induction, mirroring the sequential transformation from normal oral mucosa to OPMDs, culminating in OSCC development. By intervening during the OPMDs stage, we observed that combining PD1 blockade with photodynamic therapy (PDT) significantly mitigated oral carcinogenesis progression. Single‐cell transcriptomic sequencing unveiled microenvironmental dysregulation occurring predominantly from OPMDs to OSCC stages, fostering a tumor‐promoting milieu characterized by increased Treg proportion, heightened S100A8 expression, and decreased Fib_Igfbp5 (a specific fibroblast subtype) proportion, among others. Notably, intervening with PD1 blockade and PDT during the OPMDs stage hindered the formation of the tumor‐promoting microenvironment, resulting in decreased Treg proportion, reduced S100A8 expression, and increased Fib_Igfbp5 proportion. Moreover, combination therapy elicited a more robust treatment‐associated immune response compared with monotherapy. In essence, our findings present a novel strategy for curtailing the progression of oral carcinogenesis.

## Graphical abstract

In oral carcinogenesis, various cell types, including epithelial cells (Epi_Cxcl9) and macrophages (Macro_S100a8), exhibit significant expression of inflammatory‐related cytokines such as S100A8/S100A9. The overexpressed inflammatory cytokines recruit numerous immunosuppressive cell subsets (Tregs, Macro_Cd274, CD8_Tex, etc.). PD1 monoclonal antibody (PD1 mAb) can specifically block the functional inhibition of CD8_Tex by Macro_Cd274, but simultaneously accompanied by an increase in Treg proportion. Photodynamic therapy (PDT) not only directly kills abnormal cells but also reduces the proportion of Treg. Compared with monotherapy, the combination of PD1 mAb and PDT can reshape the microenvironment and induce a stronger therapy‐related inflammatory response, thereby preventing oral carcinogenesis.

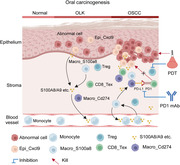

## 1 INTRODUCTION

Oral squamous cell carcinoma (OSCC) stands as the predominant subtype of head and neck squamous cell carcinoma (HNSCC), which is one of the most common cancers worldwide. In 2023, the United States is projected to have approximately 34,470 new cases of oral cancer, with an estimated 7440 fatalities resulting the disease.^1^ Oral carcinogenesis usually represents a step‐wise paradigm of carcinoma development that progresses sequentially from the normal oral mucosa, through oral potential malignant disorders (OPMDs), to OSCC. However, the mechanisms underlying the initiation and progression of oral carcinogenesis remain largely unknown, leading to a lack of effective treatment for OSCC. Therefore, understanding the dynamic features of the different stages of oral carcinogenesis is essential for the early diagnosis and treatment of OSCC.

Early treatment of malignancies is crucial to halt the process of carcinogenesis. Immune checkpoint blockade (ICB), such as pembrolizumab and nivolumab, has been approved for managing advanced HNSCC and demonstrated favorable outcomes.^2^, ^3^ One of the most common OPMDs is oral leukoplakia (OLK), with an overall reported malignant transformation rate of approximately 9.70%.^4^ Our previous work explored the efficacy of programmed cell death protein 1 (PD1) blockade in the mice model of oral carcinogenesis, demonstrating that PD1 blockade could impede the progression of oral carcinogenesis.^5^ However, we also observed that some mice showed insensitivity to PD1 blockade, which could be associated with insufficient immune cell infiltration in the microenvironment.^6^ Besides, the results of a recent nonrandomized controlled clinical trial showed that nivolumab achieved a response rate of 36% in patients with high‐risk OLK.^7^ Therefore, a combination therapy that turns lesions from “cold” to “hot” could be a potential strategy to enhance the efficacy of PD1 blockade in OLK.

Photodynamic therapy (PDT) is a minimally invasive method based on the theory that a photosensitizer can selectively accumulate in abnormal cells, and then be activated by a specific wavelength of light, triggering a series of photochemical and photobiological reactions that result in the death of abnormal cells.^8^ PDT has been used clinically for managing OLK, with complete response rates and partial response rates of 32.9 and 43.2%, respectively. However, in clinical practice, the recurrence rate of OLK after PDT ranges from 0 to 60%.^9^ Reports indicate that PDT can convert the lesion state from “cold” to “hot,” making it a potential strategy to enhance the efficacy of PD1 blockade.^10^ Currently, the combination of PD1 blockade with PDT is only used in preclinical models of solid tumors and there are no studies that have reported on the combination of PD1 blockade with PDT in OLK. Thus, it is essential to clarify the efficacy of the combination of PD1 blockade with PDT in OLK and its impact on the immune microenvironment.

Here, mice models were constructed by 4‐nitroquinoline 1‐oxide (4‐NQO) induction, which can mimic the whole process of oral carcinogenesis in humans.^11^ And whole‐exome sequencing (WES) was used to identify the mutation characteristics of this mouse model to clarify the clinical applicability of conducting research based on this animal model. Subsequently, PD1 blockade, PDT, either alone or in combination, were utilized to intervene in the OLK mice for evaluating therapeutic efficacy. Ultimately, single‐cell transcriptomic sequencing (scRNA‐seq) was conducted to uncover the composition and dynamic changes of the microenvironment that was closely associated with oral carcinogenesis, as well as to elucidate how the treatments impact the identified microenvironment composition to impede the progression of oral carcinogenesis.

## 2 RESULTS

### 2.1 The combination of PD1 blockade and PDT exerts synergistic effects in preventing the progression of oral carcinogenesis in vivo

**FIGURE 1:**
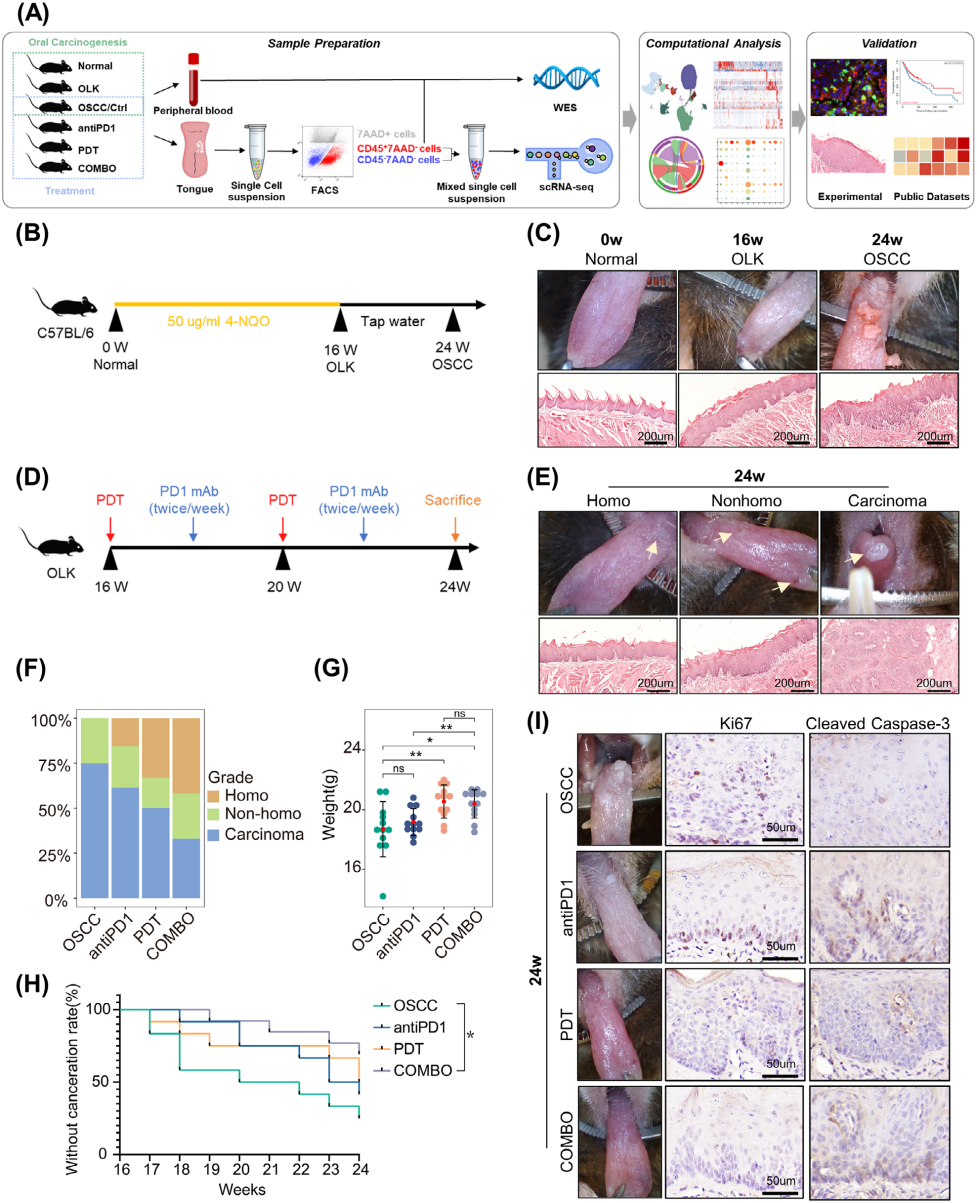
The combination of PD1 blockade and PDT exerted synergistic effects to prevent the progression of oral carcinogenesis in vivo. (A) Overview of the whole experimental strategy. The whole study could be divided into two sections: oral carcinogenesis and treatment. The dynamic features of microenvironment in oral carcinogenesis will be dissociated and how these features change in response to treatment (PD1mAb, PDT, and the combination of PD1mAb and PDT) by WES and scRNA‐seq. (B) Experimental scheme of 4‐NQO‐induced mice model. Mice were feed with 4‐NQO (50 µg/mL) in the drinking water for 16 weeks and then replaced with tap water until week 24. (C) Representative images of tongue visible lesions and H&E staining for normal, OLK, and OSCC mice. (D) Experimental scheme of treatment. OLK mice were divided into four groups randomly (each group *n* = 12): OSCC (only PBS, twice/week), antiPD1 (only PD1 mAb, twice/week), PDT (only PDT, once/month), COMBO (PD1 mAb and PDT). Because the observation endpoint of treatment is the same as the timepoint of OSCC, to avoid unnecessary mice sacrifice, OSCC mice were also regarded as controls in the section of treatment. (E) Representative images of tongue visible lesions and H&E staining for homogeneous lesions (homo), nonhomogeneous lesions (nonhomo), and carcinoma. (F) Quantification of lesion grades (homo, nonhomo, and carcinoma) from mice in the different groups (each group *n* = 12). (G) Quantification of mice weights in the different groups. Each point in the graph represents an individual mouse. ^ns^ *p* > 0.05, *p ≤ 0.05, and **p ≤ 0.01 by Wilcoxon. (H) Kaplan–Meier survival analysis across the four groups. The outcome indicator was that visible exophytic lesions were observed and the lesions’ diameter ≥1 mm (each group *n* = 12). *p ≤ 0.05 by Log‐rank test. (I) Representative images of tongue visible lesions and IHC staining of Ki67 and Cleaved Caspase‐3 in tissue slides from both control and treatment groups.

Mice models were constructed to simulate oral carcinogenesis by 4‐NQO induction (Figure 1B). To ensure the successful construction of OLK and OSCC mice models, the appearance of tongue was evaluated in each mouse at weeks 16 and 24, and three mice were randomly selected for pathological evaluation by hematoxylin and eosin (H&E) staining (Figure 1C). To determine the efficacy of the combination of PD1 blockade and PDT in preventing oral carcinogenesis, OLK mice were randomly divided into four groups: OSCC (receiving phosphate‐buffered saline [PBS]), antiPD1 (receiving PD1 mAb), PDT (receiving PDT), COMBO (receiving both PD1 mAb and PDT) (Figure 1D). Because the observation endpoint of treatment is the same as the timepoint of OSCC, to avoid unnecessary mice sacrifice, OSCC mice were also regarded as controls in the section of treatment.

To evaluate efficacy, mice weights and state of tongue lesions were measured and recorded. According to the appearance of tongues, lesions were classified into three grades: homogeneous (homo) (predominantly white, flat, thin, or wrinkled), nonhomogeneous (nonhomo) (mixed white and red, including speckled, nodular, granular, and verrucous), and carcinoma (visible exophytic lesion) (Figure 1E).^12^ Nonhomogeneous lesions typically exhibit more severe epithelial dysplasia and a higher malignant conversion rate compared with homogeneous lesions,^13^ and exophytic lesions usually indicate carcinoma. These observations are consistent with our findings (Figure 1E), supporting the use of gross appearance to assess the severity of lesions. After the intervention, the proportion of mice with homogeneous lesions increased, and more mice were under a lower malignant transformation risk in the combination treatment group than the monotherapy group (Figures 1F and S1A). Moreover, the mice in PDT and combination treatment groups had a higher weight gain compared with the OSCC group (Figure 1G). Survival analysis demonstrated that only combined treatment significantly improved mice's prognosis. And we observed better survival in the COMBO group compared with the monotherapy groups, despite the differences were not statistically significant (Figure 1H). The lack of statistical significance in the survival difference may be due to the small sample size and relatively short observation period. And the immunohistochemical results showed that compared with the control group, the expression of Ki67 decreased, and the expression of Cleaved Caspase‐3 increased in the treatment group, suggesting a decrease in proliferative capacity and an increase in apoptotic capacity of epithelial cells in the treatment group (Figure 1I). Overall, the combination of PD1 blockade and PDT can effectively prevent the progression of oral carcinogenesis, with the combined treatment exhibiting better efficacy than monotherapy.

### 2.2 Oral carcinogenesis mice models induced by 4‐NQO exhibit tobacco‐related mutational profiles

The induction of animal models using 4‐NQO is a classical method for mimicking oral carcinogenesis, yet few studies have explored its effect on somatic mutations. Here, we collected tongues and bloods coming from the six groups (normal, OLK, OSCC, antiPD1, PDT, COMBO) for WES to investigate the somatic mutational profiles. Mice tongues were dissociated into single‐cell suspensions for fluorescence‐activated cell sorting (FACS). Parts of CD45^−^7AAD^−^ cells and peripheral blood samples were used for WES.

**FIGURE 2:**
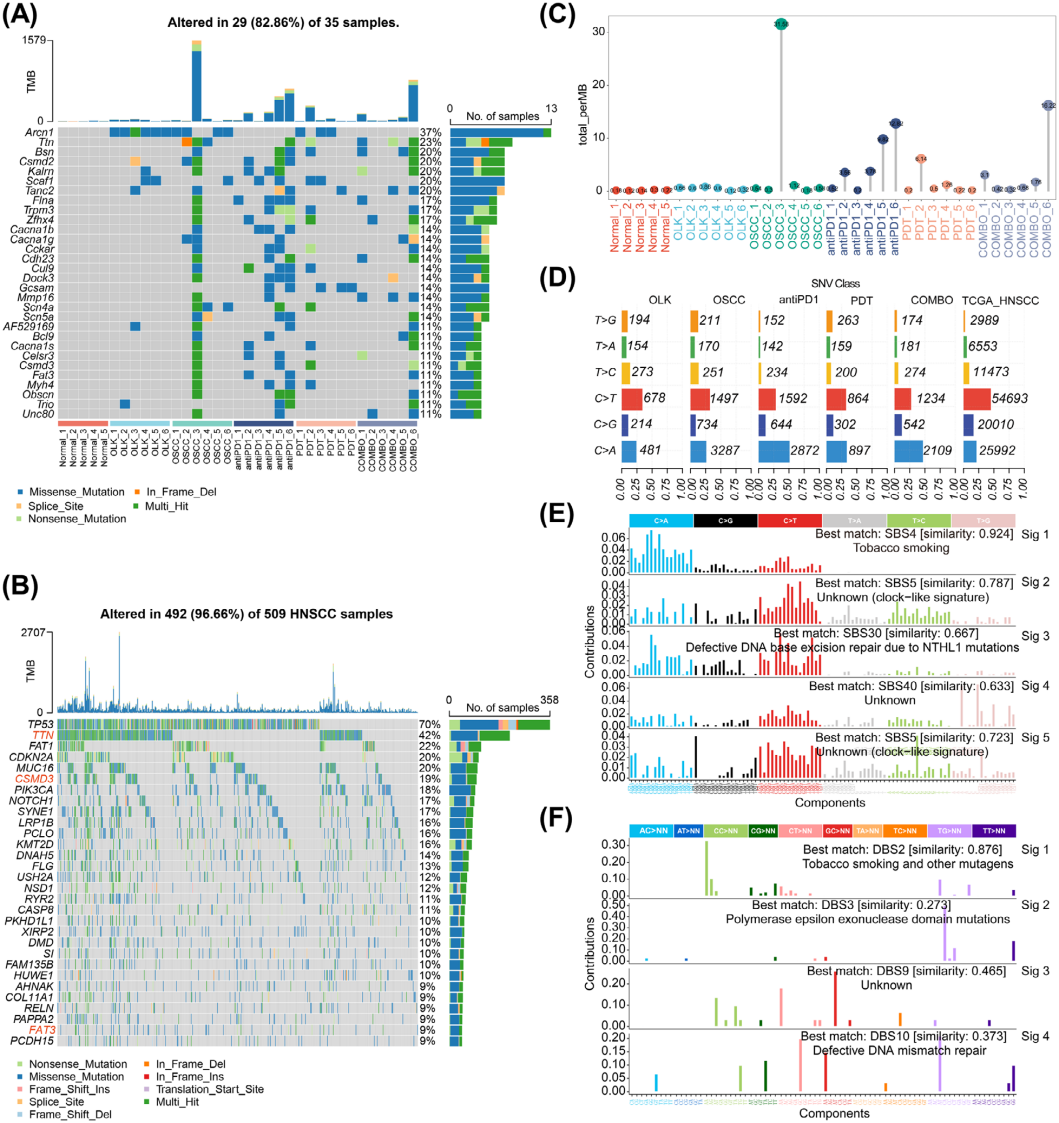
The mutational profiles of 4‐NQO‐induced oral carcinogenesis mice model were highly similar to that of smoking. (A) Oncoplot displaying the somatic landscape of mice samples. (B) Oncoplot displaying the somatic landscape of TCGA HNSCC samples. (C) The mutation burden of each mouse sample. (D) The number/percentage of single‐nucleotide variants (SNV) class in each group. (E) Signatures of single base substitution (SBS) profile. (F) Signatures of double base substitution (DBS) profile.

Oncoplot analysis revealed that missense mutations were the most frequent mutational type with the top 10 mutational genes being *Arcn1*, *Ttn*, *Bsn*, *Csmd2*, *Kalrn*, *Scaf1*, *Tanc2*, *Flna*, *Trpm3*, and *Zfhx4* (Figure 2A). Among the top 30 mutational genes, *TTN* (*Ttn*), *CSMD3* (*Csmd3*), and *FAT3* (*Fat3*) were shared mutational genes in both mice and TCGA HNSCC samples (Figure 2A,B), and they were closely associated the occurrence and progression of malignancies. The mutational burden can reflect the condition of cells’ DNA damage and repair, correlate closely with the production of neoantigens, and be used to predict the outcomes of immunotherapy in malignancies.^14^, ^15^ Our results showed that although the mutational burden increased during oral carcinogenesis, the overall level was low, with only one sample (OSCC_3) showing a higher mutational burden (31.58 per MB) (Figure 2C). The oncoplot of HNSCC also demonstrated significant heterogeneity of mutational burden among individuals (Figure 2B). Moreover, we observed the overall mutational burden increased in the treatment group (Figure 2C), indicating that PD1 blockade combined with or without PDT may induce additional mutations.

The metabolites of 4‐NQO mainly bind to guanine (G) residues resulting in DNA damage.^11^ Our results revealed the conversion of cytosine (C) to thymidine (T) or adenine (A) were the most frequent single‐nucleotide variants (SNVs) classes in both mice and HNSCC samples (Figure 2D). Deconvolution signatures of single base substitution (SBS) and double base substitution (DBS) profiles exhibited a high degree of similarity to tobacco‐related cancers, with similarities of 92.4 and 87.2%, respectively (Figure 2E,F). The above results indicate that the mouse model of oral carcinogenesis, constructed via the 4‐NQO induction, exhibits mutation patterns akin to those observed in smoking‐associated oral carcinogenesis. Consequently, our study holds implications for understanding tobacco‐related oral carcinogenesis.

### 2.3 A single‐cell atlas of oral carcinogenesis in mice tongues

**FIGURE 3:**
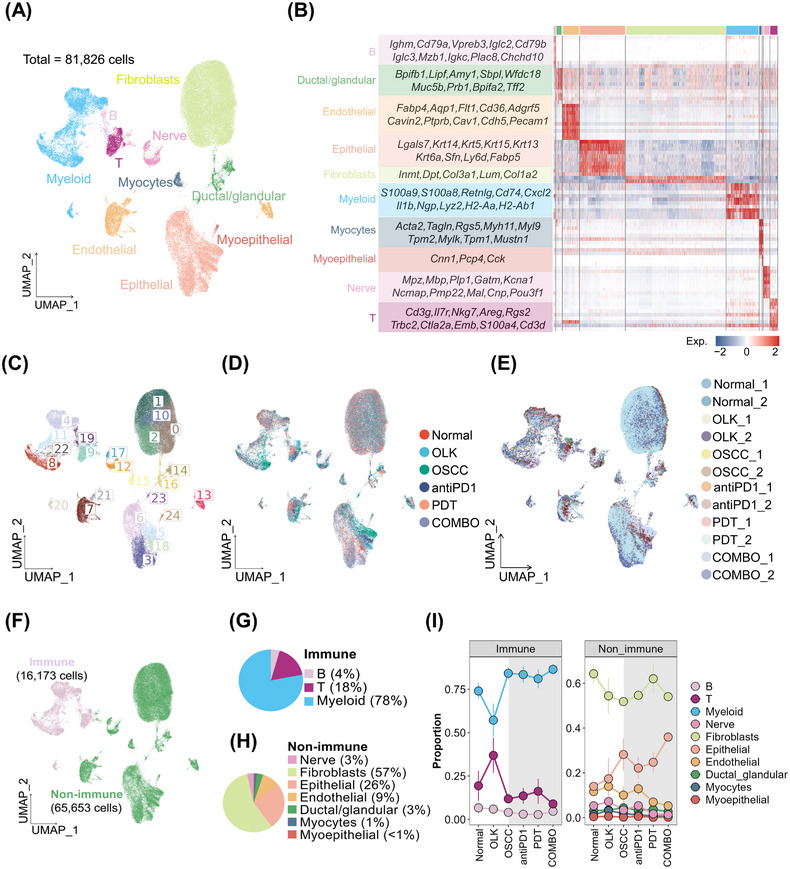
Whole‐cell atlas was mapped by scRNA‐seq. (A) UMAP plot visualizing 10 cell types encompassing 81,826 cells from all samples (*n* = 12). (B) Heatmap showing expression levels of marker genes for each cell type. (C) UMAP plot visualizing cells colored by seurat clusters (*n* = 24). (D) UMAP plot visualizing cells colored by groups (*n* = 6). (E) UMAP plot visualizing cells colored by samples (*n* = 12). (F) UMAP plot visualizing cells colored by immune cells (*n* = 16,173 cells) and nonimmune cells (*n* = 65,653 cells). (G) Pie chart showing the proportion of each immune subset in total immune cells. (H) Pie chart showing the proportion of each nonimmune subset in total nonimmune cells. (I) Line charts showing the proportion of various cell types in each group.

To explore the transcriptomic alterations occurring during different stages of oral carcinogenesis and induced by treatments (PD1 blockade combined with or without PDT), single‐cell suspensions from six groups, including normal, OLK, OSCC, antiPD1, PDT, and COMBO groups, were subjected to scRNA‐seq. CD45^−^7AAD^−^ cells and CD45^+^7AAD^−^ cells were mixed at a 4:1 ratio from three mice to minimize interindividual heterogeneity. After quality control, a total of 81,826 cells from 12 individual samples were obtained, comprising 16,173 immune cells and 65,653 nonimmune cells (Figure 3A–F and S2A–C). Based on the expression of canonical markers, immune cells were classified into myeloid cells (∼78%), T cells (∼18%), and B cells (∼4%) (Figure 3G) and nonimmune cells were classified into fibroblasts (∼57%), epithelial cells (∼26%), endothelial cells (∼9%), nerve cells (∼3%), ductal/glandular cells (∼3%), myocytes (∼1%), and myoepithelial cells (<1%) (Figure 3H). The proportion of each cell type in every group was shown in Figure 3I. To validated the similar findings in the mice samples, we also performed a reanalysis of our previous human scRNA‐seq data, which included two normal oral mucosa samples, three OLK samples, and three OSCC samples (Figure S2D,E).

### 2.4 Identification of transcriptional features of epithelial cells

**FIGURE 4:**
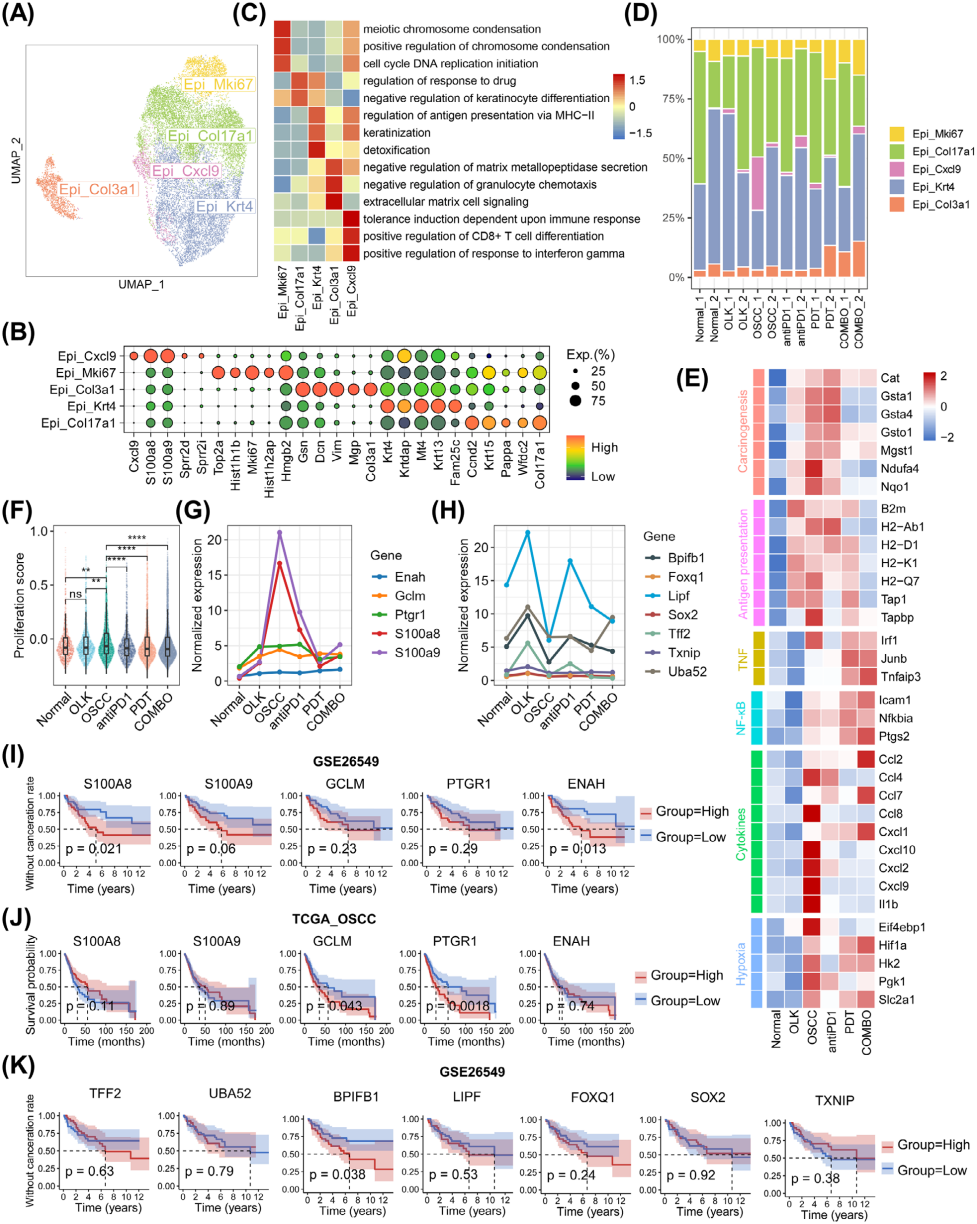
Epithelial subtypes and functional remodeling. (A) UMAP plot visualizing the identified five epithelial subtypes (total = 17,519 cells). (B) Dotplot showing expression levels of top‐expression genes for each epithelial subtype. (C) Heatmap showing the expression‐based pathway activities score of each epithelial subtype by GSVA. (D) Stacked bar plot showing the proportion of per epithelial subtype in each sample. (E) Heatmap showing expression levels of inflammatory, antigen presentation, chemical carcinogenesis, hypoxia, TNF, and NF‐κB‐related genes across the six groups. (F) Box plots showing cell proliferation‐related gene signature score of each group. Each point in the graph represents an individual epithelial cell. ^ns^ *p* > 0.05, *p ≤ 0.05, **p ≤ 0.01, ***p ≤ 0.001, and ****p ≤ 0.0001 by Wilcoxon. (G) Line charts showing normalized expression level of the genes overexpressed in the whole process in oral carcinogenesis across the six groups. (H) Line charts showing normalized expression levels of the genes only overexpressed in OLK across the six groups. (I) Kaplan–Meier survival analysis of the upregulated genes in the whole process in oral carcinogenesis in GSE26549 cohort. *p* Values were calculated by log‐rank test. (J) Kaplan–Meier survival analysis of the upregulated genes in the whole process in oral carcinogenesis in TCGA OSCC cohort. *p* Values were calculated by log‐rank test. (K) Kaplan–Meier survival analysis of the genes only overexpressed in OLK in GSE26549 cohort. *p* Values were calculated by log‐rank test.

We extracted and reclustered a total of 17,519 epithelial cells, resulting in five subtypes, namely Epi_Mki67, Epi_Col17a1, Epi_Krt4, Epi_Cxcl9, and Epi_Col3a1 (Figure 4A). We found that Epi_Mki67 exhibited elevated expression of genes (e.g., *Mki67* and *Top2a*) involved in DNA replication, reflecting the oral epithelium's routine self‐renewal ability (Figure 4B,C). Epi_Col17a1 showed increased expression of genes (e.g., *Col17a1*, *Ccnd2*, and *Krt15*) involved in the regulation of response to drug and negative regulation of keratinocyte differentiation, indicating that Epi_Col17a1 probably represents basal cells (Figure 4B,C). Epi_Krt4, with higher expression of keratinocyte marker genes (e.g., *Krt4*, *Krtdap*, and *Krt13*), likely represents keratinocytes (Figure 4B,C). Epi_Col3a1 demonstrated elevated expression of genes (e.g., *Col3a1, Gsn*, and *Dcn*) in negative regulation of matrix metallopeptidase secretion, negative regulation of granulocyte chemotaxis, and extracellular matrix (ECM) cell signaling, so Epi_ Col3a1 probably be related with the formation of the sclerotic matrix (Figure 4B,C). Epi_Cxcl9, which is essentially absent in normal samples but highly enriched in OSCC_1, represents a tumor‐specific cell type. It shows higher expression of genes (e.g., *Cxcl9*, *S100a8*, and *S100a9*) in tolerance induction, positive regulation of CD8^+^T cells differentiation, and response to interferon gamma (IFN‐γ) (Figure 4B–D).

Next, we investigated the transcriptional differences of epithelial cells among groups. Compare with normal group, the OLK and OSCC groups both featured with the upregulation of genes in chemical carcinogenesis (e.g., *Cat, Gsta1*, and *Ndufa4*) and antigen presentation (e.g., *B2m, H2‐Ab1*, and *H2‐Q7*). The expression of these genes decreased in treatment groups, especially in the COMBO group (Figure 4E). It indicated that the antigenicity of epithelial cells increased with the progression of oral carcinogenesis, but decreased when abnormal epithelial cells were eliminated by PD1 blockade with or without PDT. Furthermore, we found that genes related to tumor necrosis factor (TNF), nuclear factor kappa‐B (NF‐κB), cytokines, and hypoxia were overexpressed in the OSCC group (Figure 4E), suggesting the formation of an inflammatory and hypoxic microenvironment at the stage of OSCC. Compare with OSCC group, the PDT and COMBO groups had overexpressed genes related to TNF, NF‐κB, and hypoxia, which suggests that PDT could activate the TNF and NF‐κB pathways and aggravate hypoxia. We also found that the expression of most cytokines‐related genes (e.g., *Cxcl2*, *Ccl8*, and *Il1b*) decreased in treatment groups, except for an increased expression of *Ccl2*, *Ccl7*, and *Cxcl1* observed in COMBO group (Figure 4E). It indicated that treatments‐induced inflammatory patterns that were different from carcinogenesis‐related inflammatory patterns.

Then, we conducted analysis to determine the proliferative ability of epithelial cells. Compared with normal group, only the OSCC group exhibited an increase in epithelial proliferative ability in oral carcinogenesis. Conversely, treatment groups showed a significant decrease in epithelial proliferative ability compared with the OSCC group (Figure 4F). These results further support the notion that PD1 blockade with or without PDT can impede the excessive proliferation of epithelial cells. Additionally, our investigation revealed that specific genes such as *Enah*, *Gclm*, *Ptgr1*, *S100a8*, and *S100a9* were consistently overexpressed throughout the entire process of oral carcinogenesis, while others, including *Bpifb1*, *Foxq1*, *Lipf*, *Sox2*, *Tff2*, *Txnip*, and *Uba52*, were only overexpressed during the OLK stage (Figure 4G). Subsequently, we analyzed the relationship between these genes and prognosis in the TCGA OSCC cohort and GSE26549 (a clinical cohort of OLK). We found that the expression level of *BPIFB1*, *ENAH*, *S100A8*, and *S100A9* was negatively correlated with the prognosis of OLK, and the expression level of *PTGR1* was negatively correlated with the prognosis of OSCC (Figure 4I–K).

### 2.5 Identification of transcriptional features of stromal cells

**FIGURE 5:**
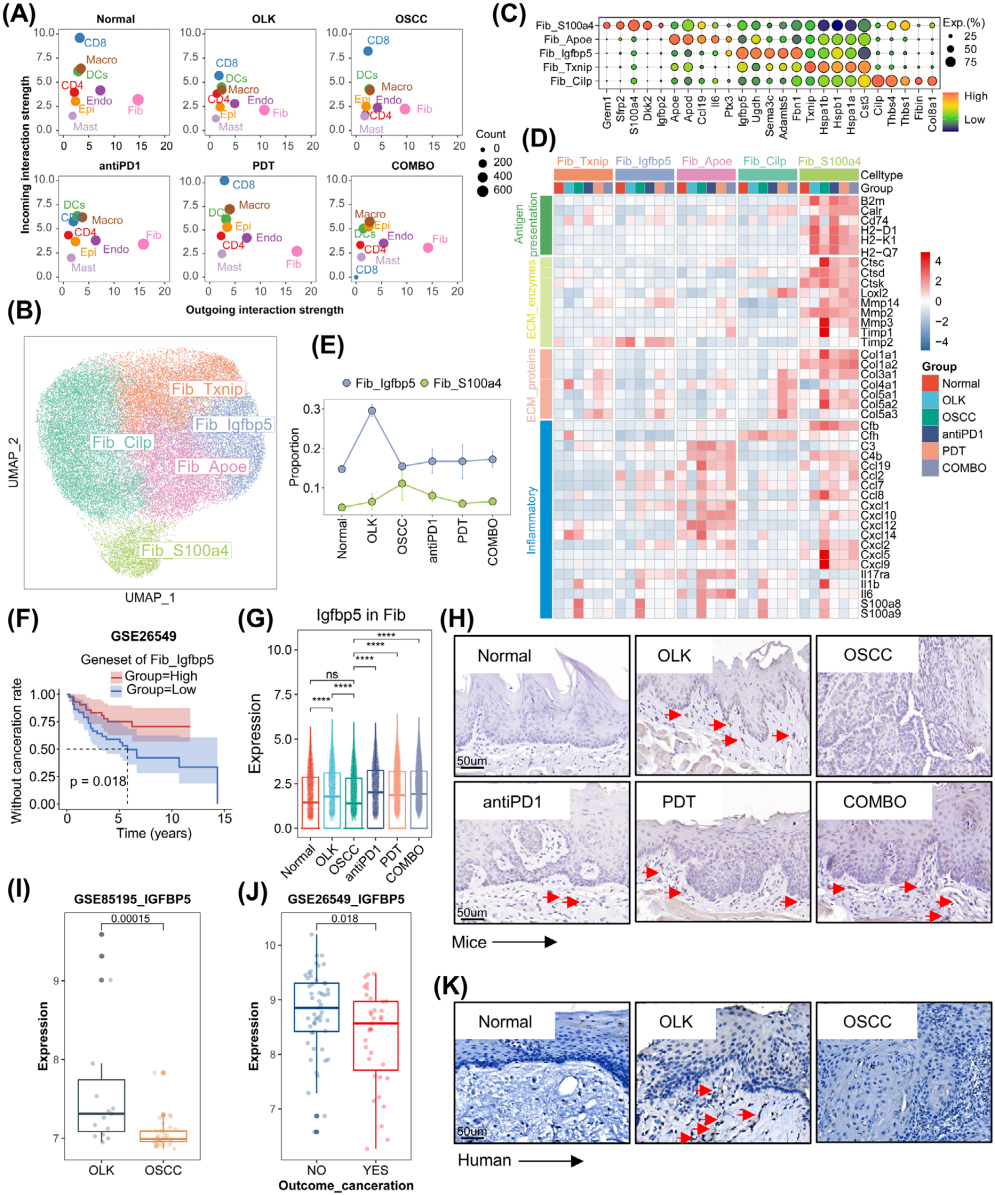
Fibroblast subtypes and functional remodeling. (A) Scatter plot visualizing the incoming/outgoing strength of each cell type scatter plot across the six groups. (B) UMAP plot visualizing the identified five fibroblast subtypes (total = 38,811 cells). (C) Dotplot showing expression levels of top‐expression genes for each fibroblast subtype. (D) Heatmap showing expression levels of antigen presentation, extracellular matrix (ECM) proteins, ECM enzymes, and inflammatory‐related genes across the six groups. (E) Folding line chart showing the proportion of Fib_Igfbp5 and Fib_S100a4 in each group. (F) Kaplan–Meier survival analysis of the genesets of Fib_Igfbp5 in GSE26549 cohort. *p* Values were calculated by log‐rank test. (G) Box plots with jitter showing the expression level of *Igfbp5* in fibroblasts across the six groups. Each point in the graph represents a fibroblast. ^ns^ *p* > 0.05, *p ≤ 0.05, **p ≤ 0.01, ***p ≤ 0.001, and ****p ≤ 0.0001 by Wilcoxon. (H) Representative images of IHC staining of IGFBP5 in mice tongue slides. (I) Box plots showing the expression level of *IGFBP5* in GSE85195. Each point in the graph represents an individual patient. The *P*‐value was calculated by Wilcoxon. (J) Box plots showing the expression level of *IGFBP5* in GSE26549. Each point in the graph represents an individual patient. The *p* Value was calculated by Wilcoxon. (K) Representative images of IHC staining of IGFBP5 in human normal oral mucosa, OLK, and OSCC tissue slides.

The results of cellular interactions indicated that, in all groups, fibroblasts had the strongest outgoing interaction strength signals and the interaction intensity between fibroblasts and other cell types in the treatment group was stronger than other groups (Figures 5A and S3A). A total of 38,519 fibroblasts were extracted from the comprehensive cell atlas and reclustered, and five subtypes were identified, including Fib_Txnip (high expression of *Txnip*, *Hspa1b*, and *Hspb1*, etc.), Fib_Igfbp5 (high expression of *Igfbp5*, *Ugdh*, and *Sema3c*, etc.), Fib_Apoe (high expression of *Apoe*, *Apod*, and *Il6*, etc.), Fib_Cilp (high expression of *Cilp*, *Thbs4*, and *Thbs1*, etc.), and Fib_S100a4 (high expression of *S100a4*, *Sfrp2*, and *S100a4*, etc.) (Figure 5B,C). Then, scores of inflammations, ECM proteins, ECM enzymes, and angiogenesis‐related genesets were calculated to infer the function state of fibroblasts’ subtypes. The results demonstrated that Fib_Apoe was characterized by the highest inflammatory effect, and Fib_Cilp was associated with angiogenesis, Fib_S100a4 was the main source of ECM proteins and enzymes and also exhibited some inflammatory effect (Figure S3B).

We found that the proportion of Fib_S100a4 increased gradually during oral carcinogenesis, and decreased after treatments (Figures 5E and S3C). Fib_S100a4 demonstrated enhanced matrix‐remodeling ability (with a higher expression of ECM proteins and enzymes‐related genes) in oral carcinogenesis (Figure 5D). The expression of ECM proteins and enzymes‐related genes also increased in human fibroblasts during oral carcinogenesis (Figure S3D,E). It suggested that ECM was in a state of lysis and remodeling in oral carcinogenesis and that Fib_S100a4 might play a vital role in this process. Besides, compared with other groups, Fib_S100a4 in OLK and antiPD1 groups had a stronger antigen presentation ability (Figure 5D). For Fib_S100a4, the expression of ECM proteins‐related genes decreased in treatments groups compared with OSCC group, however, for other cell types (Fib_Txnip, Fib_Cilp, Fib_Apoe, Fib_Igfbp5), the expression of ECM proteins‐related genes increased (Figure 5D). The inflammatory effect of fibroblasts was diminished in OLK group and significantly enhanced in OSCC group. Compared with OSCC group, the inflammatory effect of fibroblasts decreased in treatment groups (Figure 5D).

We previously identified a fibroblasts’ subtype (Fib_Igfbp5) with high expression of *Igfbp5*, and this subtype was mainly enriched in OLK group (Figure 5E). The expression level of genesets of Fib_Igfbp5 was positively correlated with the prognosis of OLK (Figure 5F). Both results of scRNA‐seq and IHC showed that the expression of *Igfbp5* (IGFBP5) was higher in the OLK group compared with the normal and OSCC groups, and in the treatment groups compared with the OSCC group (Figure 5G,H,K). In GSE85195, the expression of *IGFBP5* in OLK samples was higher than OSCC samples, and in GSE26549, OLK patients who developed cancer were with a lower baseline expression of *IGFBP5* than those who do not (Figure 5I,J).

We also examined the transcriptional features of endothelial cells, a total of 6,482 endothelial cells were reclustered. Five subtypes were identified, including Endo_Lpl (high expression of *Lpl*, *Rgcc*, and *Cd36*, etc.), Endo_Rgs5 (high expression of *Rgs5*, *Abcc9*, and *Kcnj8*, etc.), Endo_Selp (high expression of *Selp*, *Sele*, and *Ackr1*, etc.), Endo_Ccl21a (high expression of *Ccl21a*, *Mmrn1*, and *Lyve1*, etc.), Endo_Dcn (high expression of *Dcn*, *Gsn*, and *Col3a1*, etc.) (Figure S4A,C). Endo_Lpl might be capillary endothelial cells, Endo_Rgs5 might be pericytes, Endo_Selp might be venous endothelial cells, Endo_Ccl21a might be lymphatic endothelial cells, and most of Endo_Dcn might come from capillaries, and a small percentage of Endo_Dcn might come from lymphatic vessels (Figure S4B).^16^, ^17^ The proportion of per cell type in each sample were shown in Figure S4D. We found that the frequency of Endo_Selp increased in oral carcinogenesis. Compared with other cell types, Endo_Selp was with a high expression of genes (e.g., *Ackr1*, *Vwf*, *Il6*, *Vcam1*, and *Lrg1*) in leukocyte migration, cell adhesion, wound healing, and angiogenesis (Figure S4F,G). It suggested that Endo_Selp might be activated venous endothelial cells and play an important role in the regulation of immune microenvironment. Subsequently, we analyzed the differences in endothelial cells among groups. The results demonstrated that compared with normal group, the expression of inflammation‐related genes decreased in OLK group, however, increased in OSCC group. Compared with OSCC group, some inflammation‐related genes’ expression (e.g., *S100a8*, *S100a9*, *Il1b*, and *Cxcl9*) decreased in treatment groups, while some inflammation‐related genes’ expression (e.g., *Ccl2*, *Ccl7*, *Jun*, and *Fos*) increased, especially in COMBO group (Figure S4H). The different expression of inflammation‐related genes in endothelial cells could potentially contribute to the differential composition of immune microenvironment.

### 2.6 Identification of transcriptional features of immune cells

Both mice and human samples showed that immune checkpoints (*Pdcd1/PDCD1*, *Ctla4/CTLA4*, *Lag3/LAG3*, and *Havcr2/HAVCR2*) were mainly overexpressed in T cells, while their ligands (*Cd274/CD274*, *Cd86/CD86*, *Ceacam1/CEACAM1*, and *Pvr/PVR*) were mainly overexpressed in myeloid cells (Figure S5A–C). For PD‐L1 signaling pathway network, myeloid cells were the main signaling‐outcoming cell types (Figure S5D). These results indicate that myeloid cells are an important source of inhibiting T cells' function.

**FIGURE 6:**
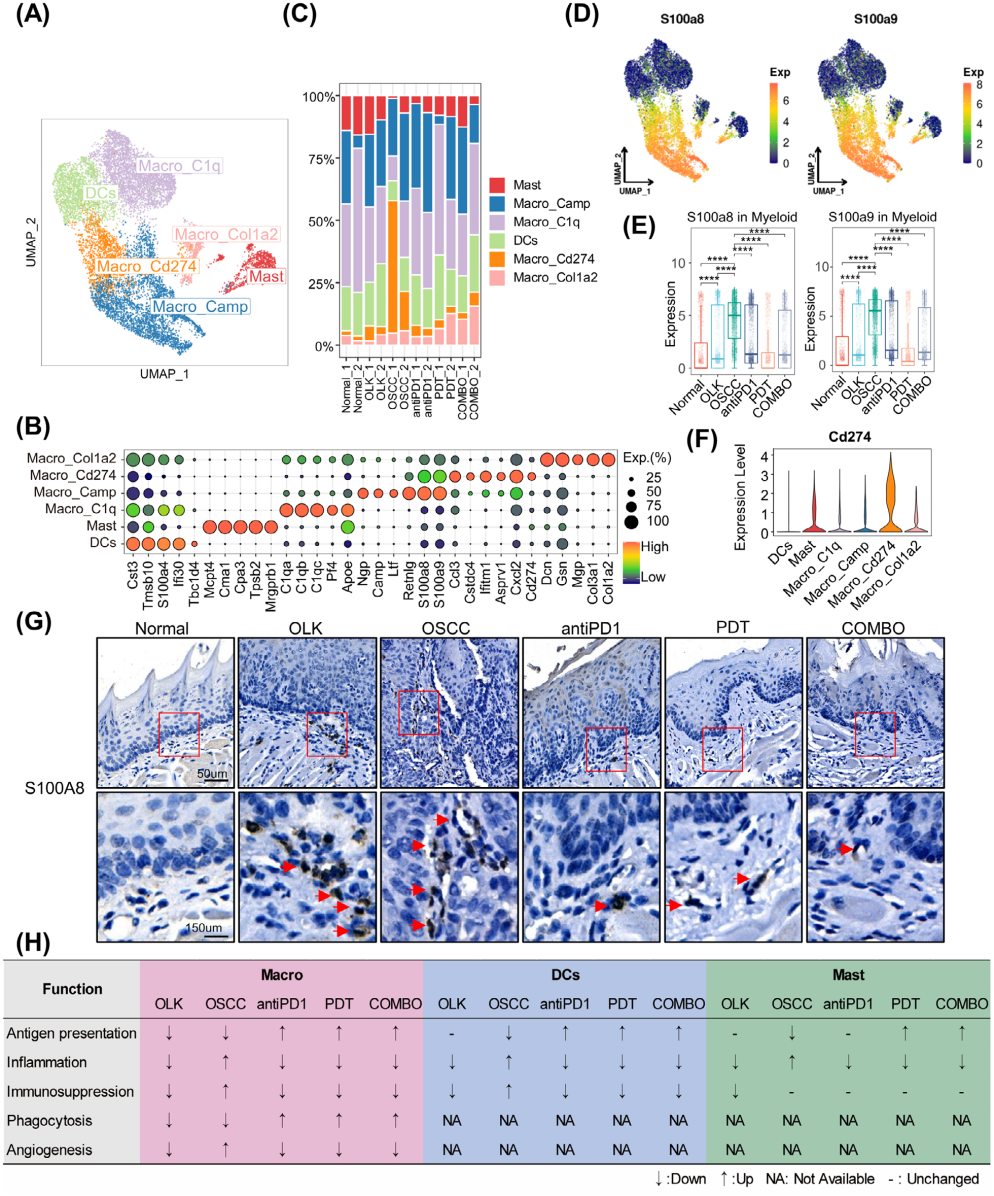
Myeloid subtypes and functional remodeling. (A) UMAP plot visualizing the identified six myeloid subtypes (total = 12,612 cells). (B) Dotplot showing expression levels of top‐expression genes for each myeloid subtype. (C) Box plot showing the proportion of per myeloid subtype in each sample. (D) UMAP of scale normalized expression of *S100a8* (left) and *S100a9* (right). (E) Box plot with jitter showing the expression level of *S100a8* (left) and *S100a9* (right) in total myeloid cells across the six groups. Each point in the graph represents a myeloid cell. ^ns^ *p* > 0.05, *p ≤ 0.05, **p ≤ 0.01, ***p ≤ 0.001, and ****p ≤ 0.0001 by Wilcoxon. (F) Violin plot showing expression levels of the *Cd274* in per myeloid subtype. (G) IHC staining of S100A8 in mice tongue slides across the six groups. (H) Summary of the main functions’ changes in DCs, macrophages, and mast cells across groups. The reference for OLK and OSCC group is normal group, and the reference for antiPD1, PDT, and COMBO is OSCC group.

Then we analyzed the transcriptional features of myeloid cells. A total of 12,612 myeloid cells were extracted and reclustered and six subtypes were identified, including four macrophages’ subtypes (Macro_C1q, Macro_Camp, Macro_Cd274, Macro_Col1a2), one mast cells’ subtype (Mast), and one dendritic cells’ subtype (DCs) (Figure 6A). The top5 overexpressed genes of per subtypes were shown in Figure 6B and the proportion of per subtype in each group was shown in Figure 6C. We found that Macro_Camp and Macro_Cd274 with a high expression of *S100a8/S100a9*, and the overall expression of *S100a8/S100a9* increased in oral carcinogenesis, while decreased in treatments groups (Figure 6D,E,G). And in human samples, the expression of *S100A8/S100A9* in macrophages also increased in oral carcinogenesis (Figure S6A). Besides, we identified a macrophages’ subtype (Macro_Cd274) with high expression of *Cd274*, and the proportion of Macro_Cd274 increased in oral carcinogenesis but decreased after treatment (Figure 6C,F). And Kyoto Encyclopedia of Genes and Genomes (KEGG) analysis revealed that the upregulated genes of Macro_Cd274 enriched in the pathway “PD‐L1 expression and PD1 checkpoint pathway in cancer” (Figure S6B). Macrophages with high expression of S100A8 and S100A9 were derived from circulating monocytes.^18^ In the presence of 4‐NQO, the inner inflammatory pathways of epithelial and stromal cells were activated, then secreted cytokines recruited a large number of peripheral circulating monocytes to infiltrate into the microenvironment. However, under the influence of microenvironment, parts of these monocytes would differentiate into macrophages with high expression of *Cd274* resulting in the functional Inhibition of T cells. We also analyzed the function state of macrophages, DCs, and Mast cells, the results were shown in Figures 6H and S6C,D. Overall, during OLK stage, myeloid cells were not completely dysfunctional with the function of angiogenesis and immunosuppression decreasing. However, during the development of OSCC, the dysfunction of myeloid cells worsened, and their protumor effect was enhanced. PD1 blockade combined with or without PDT can remodel the function of myeloid cells to enhance antitumor immunity.

Next, we extracted and reclustered T cells, identifying nine cell types among 2,840 cells, including proliferative T cells (Tprolif) (high expression of *Mki67*, *Stmn1*, and *Top2a*, etc.), exhausted CD8^+^T cells (CD8_Tex) (high expression of *Cd8b1*, *Gzmb*, and *Lag3*, etc.), effector and memory CD8^+^T cells (CD8_Tem) (high expression of *Cd8b1*, *Ccl5*, and *Ccl4*, etc.), naive CD4^+^T cells (CD4_Tnavie) (high expression of *Lef1*, *Klf2*, and *Tcf7*, etc.), regulatory T cells (Treg) (high expression of *Tnfrsf4*, *Ctla4*, and *Ikzf2*, etc.), T helper 1 like cells (Th1_like) (high expression of *Ifitm1*, *Ifitm2*, and *Ifng*, etc.), natural killer cells (NK) (high expression of *Xcl1*, *Gzmc*, and *Fcer1g*, etc.), γδT cells (γδT) (high expression of *Trdc*, *Blk*, and *Tcrg‐C1* etc.), innate lymphoid cells (ILCs) (high expression of *Il1rl1*, *Lmo4*, and *Arg1*, etc.) (Figure S7A–C). In total T cells, the ratios of CD4^+^T cells, ILCs, γδT, CD8^+^T cells, NK, and Tprolif were 32, 26, 23, 12, 3, and 3%, respectively (Figure S7D). Notably, compared with the normal group, the OLK and antiPD1 groups showed a higher proportion of Treg, particularly in the latter (Figure S7E), suggesting that increased Treg infiltration contributes to the formation of an immunosuppressive microenvironment.

We found that CD8_Tex both overexpressed with immune checkpoints and cytotoxic factors, and their exhausted score was positive with the cytotoxic score (Figure S7F,G). Meanwhile, the expression of cytotoxic genes was positive with the expression of exhausted genes in TCGA OSCC cohorts (Figure S7H). CD8_Tex and CD8_Tem were differentiated from Tprolif in response to external stimulus (Figure S7I). CD8_Tex was both qualified with exhausted and cytotoxic features, and more prone to apoptosis (Figure S7J), while CD8_Tem was both cytotoxic and inflammatory, and with a stronger proliferative ability (Figure S7K). At the OLK stage, the exhausted score only elevated in CD4^+^T cells, while at the OSCC stage, the exhausted score both elevated in CD4^+^T cells and CD8^+^T cells. After treatments, CD8^+^T cells’ exhausted score decreased, but interestingly, CD4^+^T cells’ exhausted score increased only after antiPD1 treatment (Figure S7L). It might be due to increased infiltration of Treg with high expression of *Ctla4* after PD1 blockade. Finally, we found that, during oral carcinogenesis, T cells’ proliferative score only increased in OSCC group, and compared with OSCC group, the proliferative score of T cells only increased in COMBO group. The increased proliferative capacity of T cells might serve as one of the mechanisms responsible for the synergistic antitumor effects of PD1 blockade combined with PDT treatment.

### 2.7 CXCL9 expression positively correlated with exhausted CD8^+^ T cells infiltration

**FIGURE 7:**
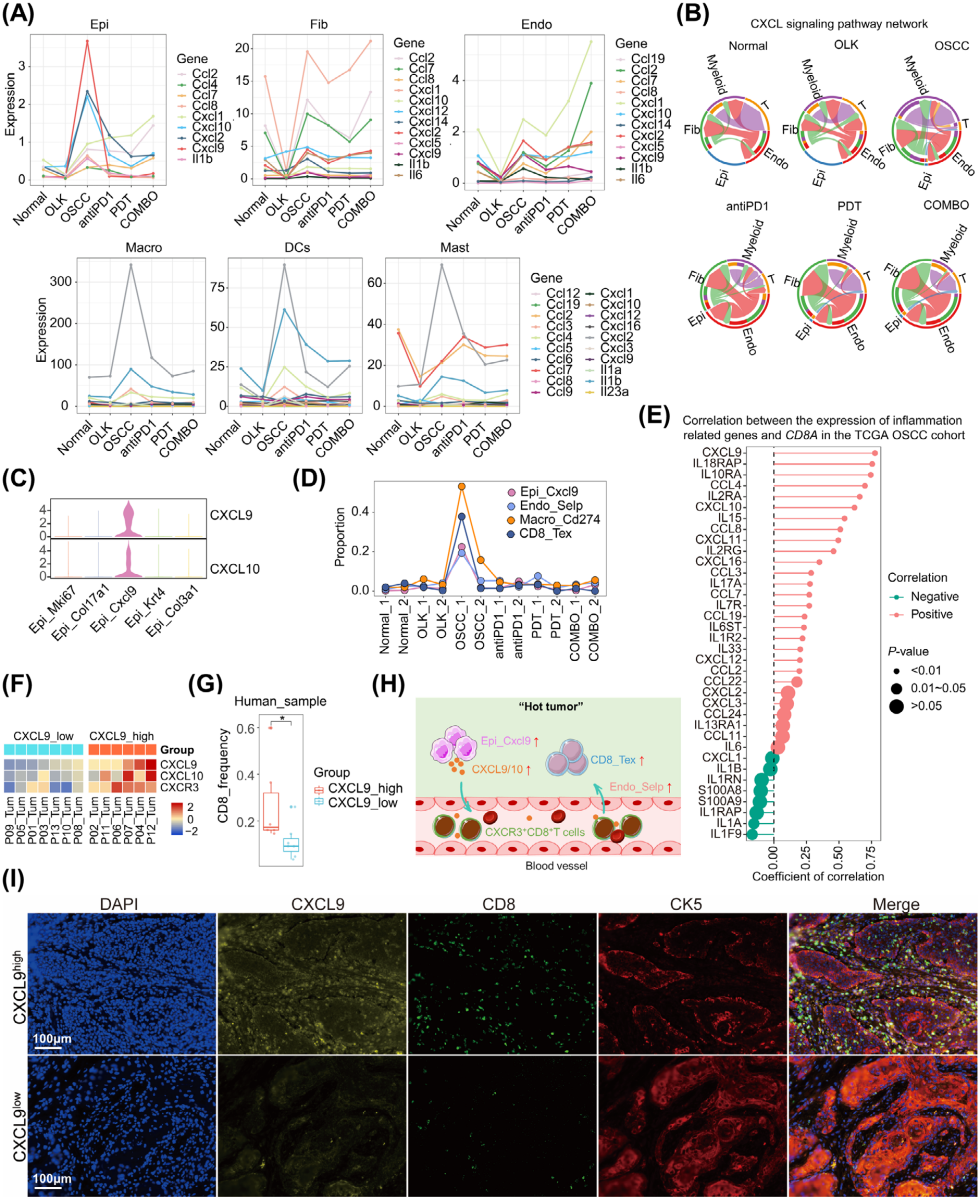
The expression of CXCL9 is positive correlated with the infiltration of exhausted CD8^+^T cells. (A) Line charts showing the expression level of inflammation‐related genes in epithelial cells, endothelial cells, fibroblasts, macrophages, DCs, and mast cells across the six groups. (B) Chord diagrams showing the strength of CXCL signaling network between cell types across the six groups. (C) Violin plot showing expression levels of the *Cxcl9* and *Cxcl10* in per epithelial subtype. (D) Line charts showing the proportion of Epi_Cxcl9, Macro_Cd274, CD8_Tex, Endo_Selp across the twelve samples. (E) Lollipop chart showing the correlation between the expression level of *CD8A* and inflammation‐related genes in TCGA OSCC cohorts. The coefficient of correlation was calculated using the Pearson correlation coefficient method. (F) Heatmap showing the expression level of *CXCL9*, *CXCL10*, and *CXCR3* of per sample in human scRNA‐seq datasets. (G) Box plots with jitter showing the frequency of CD8^+^T cells in *CXCL9* ^high^ group and *CXCL9* ^low^ group. Each jitter in the graph represents an individual patient. ^ns^ *p* > 0.05, *p ≤ 0.05, **p ≤ 0.01, ***p ≤ 0.001, and ****p ≤ 0.0001 by Wilcoxon. (H) Graphical summary of the formation of “hot tumor.” (I) mIHC staining of CXCL9, CD8, CK5 in CXCL9^high^ and CXCL9^low^ human OSCC slides.

Our study revealed that, for epithelial cells, fibroblasts, endothelial cells, and myeloid cells, the majority of inflammation‐related genes decreased in OLK group, while increased in OSCC group (Figure 7A,B). It suggested that, in oral carcinogenesis, the early stage was in an immunosuppressed state, while the late stage was in an inflammatory state. Compared with monotherapy, PD1 blockade combined with PDT could trigger stronger inflammation, and by which the combined treatments could exert synergistic antitumor effects (Figure 7A,B). In the process of data analysis, we uncovered an intriguing phenomenon involving a particular epithelial cell type (Epi_Cxcl9), which exhibited high expression levels of *Cxcl9/Cxcl10*, and Epi_Cxcl9 mainly enriched in OSCC_1 (Figure 7C,D). Recent research suggested that tumor macrophage polarity, characterized by CXCL9 and SPP1 expression rather than traditional M1 and M2 markers, exhibits a significantly strong prognostic correlation in HNSCC.^19^ In our data, besides epithelial cells, myeloid cells also express *Cxcl9* (Figure S8A). Furthermore, *Cxcl9* and *Spp1* showed mutually exclusive expression in macrophages (Figure S8B–D). And consistent with the results in epithelial cells, *Cxcl9* was predominantly highly expressed in the OSCC_1 sample and relatively low in other samples (Figure S8E).

Besides, OSCC_1 was with a higher infiltration of CD8_Tex, Macro_Cd274, and Endo_Selp (Figure 7D). We assumed that the expression of CXCL9 was positively correlated with the infiltration of CD8^+^T cells, and then we confirmed our hypothesis in public datasets and human OSCC samples. In TCGA OSCC cohorts, the expression of *CD8A* was positively correlated with the expression of *CXCL9* (Figure 7E). We had also analyzed the expression of *CXCL9* with the infiltration of CD8^+^T cells in human OSCC scRNA‐seq datasets. The OSCC samples were divided into *CXCL9* ^high^ and *CXCL9* ^low^ groups according to the expression of *CXCL9*, and the results demonstrated that *CXCL9* ^high^ group with a higher frequency of CD8^+^T cells (Figure 7F,G). The results of multiplex immunohistochemistry (mIHC) also revealed that CXCL9^high^ sample with a higher infiltration of CD8^+^T cells in human OSCC slides (Figure 7I). In mice tongue slides, we observed that CXCL9 was predominantly highly expressed in the OSCC stage during oral carcinogenesis. The treatment group showed lower expression of CXCL9 compared with the OSCC group (Figure S8F). Based on the above findings, the expression of CXCL9 was positively correlated with the infiltration of exhausted CD8^+^T cells, which contributed to the formation of “hot” tumors (Figure 7H).

## 3 DISCUSSION

In this study, we employed scRNA‐seq and WES to comprehensively identify dynamic transcriptional features of microenvironment and evolutionary features of mutation in oral carcinogenesis. And we also clarified the synergistic efficacy of the combination of PD1 blockade and PDT in OLK mice, and identified the alterations of microenvironment induced by these treatments. Our findings offer novel insights into the mechanisms driving oral carcinogenesis, as well as a new strategy for preventing its progression.

The mouse model induced by 4‐NQO could mimic the whole process of oral carcinogenesis and the metabolite of 4‐NQO bound mainly to the residues of G.^11^ Our results revealed that somatic SNVs primarily manifested as C>T and C>A substitutions, consistent with previous reports. While there exist disparities between the somatic mutation characteristics observed in this mouse model and the overall mutation patterns observed in the HNSCC cohort from TCGA due to species‐specific genetic background distinctions, both SBS and DBS profiles underscored that the 4‐NQO‐induced mouse model of oral carcinogenesis displayed mutational profiles reminiscent of tobacco exposure. Furthermore, the 4MOSC cell line, derived from 4‐NQO‐induced HNSCC mice model, also exhibited mutational profiles characteristic of tobacco exposure.^20^ Hence, our findings hold significant implications for understanding tobacco‐related precancerous conditions and malignancies.

We found that microenvironmental disruption in oral carcinogenesis primarily occurs from OLK to OSCC stages. Both the microenvironment of OLK and OSCC have immunosuppressive characteristics, but the extent of immune cell infiltration differs between the two.^21^ OLK is characterized by lower immune cell infiltration, whereas OSCC is associated with infiltrated immune cells exhibiting immunosuppressive phenotypes. Studies have shown that PD1 blockade can remodel the phenotypes of suppressive immune cells, while PDT can induce a “hot” response in the tumor.^8^, ^22^, ^23^ Our study confirmed that the combination of PD1 blockade with PDT significantly prevented the progression of oral carcinogenesis. Unlike cancer‐associated inflammation, treatment‐induced acute inflammation has been shown to have antitumor effects.^24^ Our work also demonstrated that treatments‐induced inflammatory patterns were different from those observed in the OLK and OSCC groups. Specifically, the proportion of immunosuppressive cell types (e.g., CD8_Tex and Macro_Cd274) decreased, and the antitumor function of myeloid cells, such as antigen presentation and phagocytosis, was enhanced after treatments. Additionally, we observed an increased proportion of Tregs during oral carcinogenesis. Furthermore, PD1 mAb treatment led to a further increase in Tregs. This phenomenon could be attributed to compensatory Treg proliferation resulting from incomplete depletion included by ICB.^25^ In an animal model of renal cell carcinoma, the Treg proportion decreased after PD1 mAb combined with vascular‐targeted PDT compared with monotreatment.^26^ A similar phenomenon was observed in our study. Moreover, in comparison with individual treatments, the combination of PD1 blockade and PDT triggered a more potent antitumor immune response, elucidating the synergistic antitumor effects of combination therapy.

The microenvironment of OSCC displayed robust inflammatory features, characterized by numerous cell types exhibiting high expression of inflammatory‐related genes. Particularly noteworthy was the identification of an inflammatory epithelial subtype marked by elevated expression of Cxcl9 (referred to as Epi_Cxcl9). Epi_Cxcl9 was predominantly enriched in OSCC samples, with minimal presence in normal samples, indicating its close association with oral carcinogenesis under the influence of 4‐NQO. Moreover, the proportion of Epi_Cxcl9 decreased following intervention with PD1 mAb, either alone or in combination with PDT. Subsequent investigations revealed a positive correlation between CXCL9 expression and the infiltration of exhausted CD8^+^T cells. The upregulation of CXCL9 contributed significantly to the formation of “hot” tumors, characterized by heightened immune cell infiltration and activity within the tumor microenvironment.^27^, ^28^ Myeloid cells have been identified as significant sources of immunosuppressive effects in HNSCC.^29^, ^30^ Our results corroborate this notion, demonstrating that the increased infiltration of exhausted CD8^+^T cells coincided with an increase in the presence of immunosuppressive macrophages (designated as Macro_Cd274). Notably, myeloid cells possess the capability to inhibit T cells’ function by PD‐L1/PD1 signaling pathway. Previous results suggested that Epi_Cxcl9 plays a role in shaping an immunosuppressive microenvironment. However, early intervention with a combination of PD1 mAb and PDT during the oral precancerous stage can mitigate the proportion of Epi_Cxcl9, thereby restraining the development of an immunosuppressive microenvironment.

ECM was an essential component of microenvironment and had a close link with the development of malignancies.^31^ We identified a fibroblasts’ subtype (Fib_S100a4) with high expression of ECM‐related proteins and enzymes, and the proportion of Fib_S100a4 increased constantly in oral carcinogenesis. In pancreatic cancer, the prognosis of patients with high expression of ECM enzymes‐cleaved collagens I was worse than patients with high expression of intact collagens I.^32^ Our study suggested that the ECM was in a state of lysis in oral carcinogenesis, and Fib_S100a4 might be responsible for the remodeling of ECM; however, the specific roles and mechanisms of Fib_S100a4 in oral carcinogenesis should be future studied. Although the proportion of Fib_S100a4 decreased after treatments, other fibroblasts’ subtypes were with higher expression of collagen‐related genes, which might result in the sclerosis of ECM. The sclerotic ECM could prevent the penetration of drugs and immune cells into the deeper layers of lesions, weakening the effectiveness of treatments.^33^, ^34^, ^35^ The deposition of collagen in treatments groups might be associated with the development of secondary treatment resistance in abnormal cells. Furthermore, the high expression of collagen in PDT and COMBO groups might also be associated with scar healing.

Early detection, diagnosis, and treatment were the keys for cancer management. Here, we aimed to discuss some unique findings in OLK. We identified a fibroblast cell type with high expression of *Igfbp5*, which was mainly enriched in OLK. Our analysis of human OLK cohorts found that overexpressed *IGFBP5* was correlated with a better prognosis. IGFBP5 was a member of the insulin‐like growth factor (IGF) binding protein family and played a role in regulating cell differentiation, proliferation, apoptosis, and adhesion.^36^ IGFBP5 could regulate the differentiation of oral keratinocytes and IGFBP5 deficiency might be involved in the malignant transformation of oral keratinocytes.^37^ The protective role of Fib_Igfbp5 in OLK may be attributed to the oversecreted IGFBP5, which competitively binds IGF with insulin‐like growth factor receptor (IGFR), thereby weakening the growth factor‐like effects triggered by IGF–IGFR axis activation and inhibiting excessive proliferation of epithelial cells. However, this hypothesis needed further experimental design in the future to provide further evidence. The previous results reminded that IGFBP5 might act as the role of “firefighters” in oral carcinogenesis. Interestingly, the expression of IGFBP5 increases after treatment with PD1 mAb combined with or without PDT, indicating that the aforementioned treatment measures could provide a protective effect for ‘firefighters’ in oral carcinogenesis. We also found that *S100a8/S100a9* were overexpressed in the whole process of oral carcinogenesis. However, only in OLK, the overexpressed *S100A8/S100A9* were correlated with a worse prognosis. S100A8/S100A9 belong to the calcium‐binding protein family. Because of their strong inflammatory effects (recruit myeloid cells, enhance myeloid cell chemotaxis and adhesion, etc.), they often were described as cytokine‐like proteins.^38^, ^39^ S100A8/S100A9 mainly expressed in myeloid cells.^38^ Our study also showed that S100A8/S100A9 mainly highly expressed in myeloid cells. And in other cell types (epithelial cells, endothelial cells, fibroblasts), the expression of S100A8/S100A9 also increased in oral carcinogenesis while decreased after treatments. Previous studies have reported that the overexpressed S100A8/S100A9 in tumor tissues is closely related to tumor‐associated inflammation, epithelial–mesenchymal transition, and metastasis, contributing to poor prognosis.^40^, ^41^, ^42^, ^43^ However, the role of S100A8/S100A9 in oral carcinogenesis remains relatively unexplored. Our findings suggest that S100A8/S100A9 act as tumor‐promoting inflammatory cytokines and may serve as prognostic indicators for OLK. Interventions with PD1 mAb, PDT, or their combination led to decreased expression of *S100a8/S100a9*. This underscores the potential of intervening during the precancerous stage to mitigate the effects of tumor‐promoting inflammatory factors in the microenvironment and consequently delay the progression of oral carcinogenesis.

In summary, our study revealed that the combination of PD1 mAb and PDT holds promise in impeding the progression of oral carcinogenesis by modulating the composition of the microenvironment. Moreover, this combination therapy elicits more robust treatment‐related immune responses compared with monotherapy, thereby exerting a synergistic therapeutic effect. Based on these findings, we emphasized the significance and imperative nature of early intervention and combined treatments in the clinical management of oral carcinogenesis. However, our study has some limitations. The small sample size and short observation period prevented us from detecting a statistically significant difference in survival between the COMBO group and the monotherapy group, despite a visible trend suggesting improved outcomes with the combination therapy. Future studies with larger number size and extended periods are necessary to validate our findings and fully elucidate the long‐term benefits associated with this promising therapeutic approach. Additionally, our study employs a descriptive research approach to characterize the overall remodeling features of the microenvironment using multiomics techniques, thereby highlighting significant findings for future mechanistic research.

## 4 MATERIALS AND METHODS

### 4.1 Mice

Female C57BL/6 mice, aged from 4 to 6 weeks (*n* = 74), were obtained from Beijing Hua Fukang Animal Laboratory Company. Four to five mice were allocated to the same cage for feeding. The mice were given with tap water containing 50 µg/mL 4‐NQO (Sigma–Aldrich) for 16 weeks, followed by plain tap water. To ensure successful OLK and OSCC mouse model development, the gross alterations of the tongue were evaluated in each mouse at 16 and 22 weeks, and three mice were randomly selected for pathological evaluation by H&E staining. We also collected major visceral organs from OLK and OSCC mice for H&E staining. The H&E staining revealed significant pathological changes in the esophageal tissues of both OLK and OSCC mice, while no apparent abnormalities in the lungs, spleen, heart, liver, and kidney tissues (Figure S1B). The study protocols were approved and conducted in accordance with the guidelines set forth by the West China Hospital of Stomatology Institutional Review Board (WCSHIRB‐D‐2020‐331).

### 4.2 Treatment

Each mouse was labeled with an ear tag, and then randomized into four groups (12 mice per group) using the “randomizr” package in R software^44^ based on the ear tag numbers. To ensure the reliability of the experimental results, only the intervention personnel were aware of the specific grouping of the mice, while the researchers responsible for efficacy evaluation and statistical analysis were blinded to the specific group assignments. The mice from the control group were treated with 1× PBS (Biosharp) (200 µg per mouse, twice per week) by intraperitoneal injection. The mice from the antiPD1 group were treated with PD1 monoclonal antibody (RMP1‐14; BioXCell) (200 µg per mouse, twice per week) by intraperitoneal injection. The mice from PDT group were treated with PDT (once per month). And the mice from COMBO were both intervened with PD1 monoclonal antibody and PDT. Furthermore, tongues of mice in the control group and the antiPD1 group were locally injected with 10 µL of 1× PBS.

The details for PDT are as follows. Mice were anesthetized with Zoletil (Virbac), then tongues were topically injected with 10 mL 20% 5‐aminolevulinic acid (Shanghai Fudan‐zhangjiang). After topical injection about 1 h, the whole tongue was exposed to 632.8 nm red LED light for 3 min and the output power is 5 mW.

Finally, mice were sacrificed after eight weeks of intervention. The weights of mice were measured with a scale and the status of tongue lesions was evaluated. The survival outcome indicator was that visible exophytic lesions were observed and the diameter ≥1 mm. To ensure the reliability of the experimental results, the experimenters responsible for outcome measurements were blinded to the experimental group allocation. Mice tongues and blood samples were collected for the subsequent experiments.

### 4.3 Immunohistochemistry

Mice tongue tissues embedded in paraffin were subjected to immunohistochemical staining using antibodies against Ki67 (28074‐1‐AP; Proteintech), Cleaved Caspase‐3 (9664S; Cell Signaling Technology), IGFBP5 (55205‐1‐AP; Proteintech), and S100A8 (66853‐1‐Ig; Proteintech). The immunostaining was performed using the DAB Detection Kit (Gene Tech) following the instructions.

### 4.4 H&E staining

The paraffin‐embedded tissues of mouse tongue, esophagus, lung, heart, spleen, liver, and kidney tissues were processed for H&E staining following the protocol of the Hematoxylin‐Eosin Stain Kit (G1120; Solarbio). And the tongue lesions were then independently assessed by two pathologists.

### 4.5 Multiplex immunohistochemistry

The paraffin‐embedded human normal oral mucosa, OLK, and OSCC tissues were obtained from the West China Hospital of Stomatology, Sichuan University. All specimens underwent histological diagnosis by pathologists. Written informed consent was provided by all included patients, and this study was approved by the West China Hospital of Stomatology Institutional Review Board (WCSHIRB‐D‐2020‐277).

Human tissue and mice tongue slides were stained using the PerkinElmer OPAL 7‐Colour Automation IHC kit and the following antibodies: anti‐Cytokeratin 5 (ab52635; Abcam), anti‐CD8 (ab4055, ab237723; Abcam), anti‐CXCL9 (22355‐1‐AP; Proteintech). Nuclei were stained with 4′,6‐diamidino‐2‐phenylindole. The experiment was conducted following the mIHC kit instructions. Immunofluorescence slides were scanned and analyzed using the Vectra 3.0 and images were visualized in Phenochart v.1.0.8 (AKOYA Bioscience).

### 4.6 Single‐cell suspension preparation and FACS

Tongues (all groups were six mice, except for normal group which was five mice) coming from different groups were dissected, minced, and resuspended in a digestion medium containing HEPES Solution (C0217‐500 mL; Beyotime Biotechnology), 4U/mL Dispase (07913; Stem Cell), 2 µg/µL DNAse I (DN25; Sigma), and 5 µg/µL Collagenase Type II (LS004176; Worthington) and incubated at 37°C for 60 min with shaking to obtain single‐cell suspensions. Red blood cell lysate (Beyotime) was used to lyse red blood cells, and then the suspensions were incubated with FITC Rat Anti‐Mouse CD45 (553080; BD Biosciences) and 7AAD (559925; BD Biosciences) for 30 min at room temperature, then washed with 1× PBS and filtered through 40 µm filters. The stained cells were sorted by WOLF (NanoCellect). CD45^+^7AAD^−^cells and CD45^−^7AAD^−^cells were collected for the next experiments.

### 4.7 Whole‐exome sequencing

Two or three mice's blood coming from the same group were mixed into one sample. The collected CD45^−^7AAD^−^cells and blood were used to construct libraries using the Agilent liquid capture system (Agilent SureSelect Human All Exon V6) according to the manufacturer's instructions. The DNA libraries were sequenced using Illumina novaseq 6000 system, resulting in 150 bp paired‐end reads. Raw sequencing data were aligned to the mm10 reference genome using by BurrowsWheeler Aligner (BWA) software to obtain the original mapping results in BAM format.^45^ Somatic mutations were identified by comparing the sequencing data from each tongue sample with that from peripheral blood samples (all mice were genetically identical). Subsequently, Samtools^46^ and Sambamba were respectively employed to sort bam files and mark duplicates to generate the final BAM file. If a read or a pair of reads has multiple mapping positions, the strategy adopted by BWA are to select the best one, if there are multi best mapping position, we randomly pick one. The mapping step is challenging due to mismatches, which may include true mutations and sequencing errors, as well as duplicates resulting from PCR amplification. These duplicate reads are uninformative and should not be considered as evidence for variants. Sambamba is used to mark these duplicates for subsequent exclusion from the analysis. SAM tools mpileup and bcftools were utilized for variant calling and identification of SNP and InDels. The somatic SNV was detected by muTect,^47^ the somatic InDel by Strelka,^48^ and Control‐FREEC^49^ was used to detect somatic CNV. ANNOVAR^50^ is performed to do annotation for VCF (Variant Call Format) file obtained in the previous step. At this step, variant position, type, conservative prediction and other information are obtained from various databases, such as dbSNP, 1000 Genome, esp6500, GnomAD, CADD, HGMD, COSMIC, and so on. Mutational signatures were extracted and analyzed using the R package sigminer^51^ and Maftools.^52^ WES data of HNSCC were downloaded using the R package TCGAmutations.^53^

### 4.8 scRNA‐seq and data analysis

CD45^+^7AAD^−^cells and CD45^−^7AAD^−^cells from the same mouse were mixed at the ratio of about 1:4. To reduce the heterogeneity of individuals, two or three mice's single‐cell suspensions from the same group were mixed into one sample. At last, a total of 12 samples (each group *n* = 2) were used for scRNA‐seq. Individual cells were sorted and captured respectively in nanoliter droplets using Chromium (10× Genomics). scRNA‐seq libraries were prepared using Chromium Single Cell 5′ Reagent Kits (10× Genomics) following the manufacturer's instruction. They were sequenced on Illumina Novaseq platform and mapped to the mice reference genome (mm10) using CellRanger. The raw gene expression matrices obtained from CellRanger were converted to Seurat objects using the R package Seurat.^54^ Genes detected in fewer than 10 cells were filtered. Cells with over 500 genes, fewer than 5% mitochondrial genes, and unique molecular identifiers from 1000 to 20,000 were retained. A total of 81,826 high‐quality cells were retained for further analysis. Subsequently, the filtered Seurat object was normalized and scaled. The principal components were computed by the function “RunPCA.” Using “ElbowPlot” to select appropriate principal components. Cell clusters were identified using “FindCluster” with resolutions ranging from 0.1 to 2 for different cell types. UMAP visualization was performed by using “RunUMAP.” Detailed classification information for different cell types is provided in Table S1.

### 4.9 Marker gene identification and cell annotation

“FindAllMarkersMAESTRO” from the R package MAESTRO^55^ was utilized to identify the differential expression genes (DEGs) within each cluster, and cell clusters were annotated using canonical marker genes.

### 4.10 Gene set variation analysis

The R package GSVA^56^ was employed to compute the pathway activity of each subset using default parameters. The genesets from the KEGG and Gene Ontology (GO) were acquired utilizing the msigdbr package.^57^ Visualization of the results was performed using the Pheatmap package.^58^

### 4.11 Cell transition trajectory and diffusion map analysis

Monocle 2^59^ was utilized for the trajectory analysis to identify the developmental origins of CD8^+^T cells. DEGs were identified by “differentialGeneTest,” and then the DEGs have been employed in cell ordering. Following data dimensionality reduction, the pseudo‐time trajectory of CD8^+^T cells was constructed using default parameters.

### 4.12 Gene signature score

The “AddModuleScore” function from Seurat package was employed to evaluate functional activity. Genesets utilized for computing gene signatures in myeloid cells were retrieved from the MsigDB database. Table S2 presents detailed information of other genesets. Statistical significance was assessed using the Kruskal–Walli's test with *p* value adjustment by the Benjamini–Hochberg method. Statistical significance was denoted by asterisks as follows: *p value < 0.05, **p value < 0.01, ***p value < 0.001, ****p value < 0.0001.

### 4.13 GO and KEGG enrichment analysis

The “FindMarkers” function of the Seurat package was used to identify DEGs among groups. Upregulated DEGs (avg_log2FC > 0.3 and p_val_adj < 0.05) were used to GO analysis using “enrichGO” function or KEGG analysis using the “enrichKEGG” function of the clusterProfiler package^60^ with default parameters.

### 4.14 Interaction between cell types

CellChat package^61^ was used to reveal interaction between cell types with default arguments.

### 4.15 Public datasets

GSE26549 and GSE85195 were downloaded from GEO (Gene Expression Omnibus, NCBI), bulk RNA‐seq of human OSCC datasets were downloaded from TCGA (The Cancer Genome Atlas—NCI), and human scRNA‐seq datasets were downloaded from GSA‐human (Genome Sequence Archive for Human, National Genomics Data Center of China). Survminer package^62^ was used for survival analysis. corrplot package^63^, ^64^ was used for genes expression correlations analysis.

### 4.16 Data visualization

ggplot2,^65^ ggpubr,^66^ and pheatmap^58^ were used to visualize results. stat_compare_means function from ggpubr package was used to calculate the *p* value between groups. *p value < 0.05, **p value < 0.01, ***p value < 0.001, ****p value < 0.0001.

## AUTHOR CONTRIBUTIONS

Y. D. and K. Z. contributed to manuscript drafting, data acquisition, and analysis. H. D., N. J., J. L., X. Z., X. J., and Q. C. provided material and technological support. R. A., C. Z., and F. M. conducted the assessment of tongue images. Y. Z. and T. L. were involved in manuscript revision, proofreading, study conceptualization and design, funding acquisition, and study supervision. Y. D. and K. Z. made equal contributions. Correspondence should be addressed to Y. Z, and T. L.. All authors have read and approved the final manuscript.

## CONFLICT OF INTEREST STATEMENT

All authors disclosed no relevant relationships.

## ETHICS STATEMENT AND CONSENT TO PARTICIPATE

The study protocols were approved and performed following the guidelines of the West China Hospital of Stomatology Institutional Review Board (WCSHIRB‐D‐2020‐331).

## Supporting information

Supporting Information

## Data Availability

The raw sequence data of mice scRNA‐seq and WES in this study have been deposited in the Genome Sequence Archive of the BIG Data Center at the Beijing Institute of Genomics, Chinese Academy of Science, under the accession number GSA: CRA007661 (accessible at https://ngdc.cncb.ac.cn/gsa), and GSA‐human: HRA004340 (accessible at https://ngdc.cncb.ac.cn/gsa‐human). GSE26549 and GSE85195 were obtained from the GEO (https://www.ncbi.nlm.nih.gov/geo/). TCGA OSCC cohorts were obtained from the TCGA database (https://www.cancer.gov/ccg/research/genome‐sequencing/tcga). All other relevant data are available from the corresponding author on request.
