# Supplementary material for: Single‐cell transcriptome dissecting the microenvironment remodeled by PD1 blockade combined with photodynamic therapy in a mouse model of oral carcinogenesis
Source: MedComm (2020). 2024 Jul 2;5(7):e636. doi: 10.1002/mco2.636 (PMC11220179; doi:10.1002/mco2.636)
Supplement: Supplementary file 1 — Supporting Information [file MCO2-5-e636-s001.pdf]

Single-cell Transcriptome Dissecting the Microenvironment Remodeled by PD1 blockade Combined with Photodynamic Therapy in a Mouse Model of Oral Carcinogenesis

Yunmei Dong[1,2,#], Kan Zeng[1,#], Ruixue Ai[1], Chengli Zhang[1], Fei Mao[1], Hongxia Dan[1], Xin Zeng[1], Ning Ji[1], Jing Li[1], Xin Jin[2], Qianming Chen[1], Yu Zhou[1,3,*], Taiwen Li[1,4,*]

[1] State Key Laboratory of Oral Diseases, National Clinical Research Center for Oral Diseases, Chinese Academy of Medical Sciences Research Unit of Oral Carcinogenesis and Management, West China Hospital of Stomatology, Sichuan University, 610041, Chengdu, China
[2] Chongqing Key Laboratory of Oral Diseases and Biomedical Sciences, Chongqing, China
[3] State Institute of Drug/Medical Device Clinical Trial, West China Hospital of Stomatology, 610041, Chengdu, China
[4] Collaborative Innovation Center for Cancer Personalized Medicine, Nanjing Medical University, Nanjing, China

[#] The authors contributed equally to this study and share first authorship.
* Corresponding authors: Taiwen Li and Yu Zhou
State Key Laboratory of Oral Diseases, National Clinical Research Center for Oral Diseases, Chinese Academy of Medical Sciences Research Unit of Oral Carcinogenesis and Management, West China Hospital of Stomatology, Sichuan University, No. 14, Section 3, Renmin South Road, Chengdu 610041, China. Tel: +86 15982367077; Fax: +86 028-85501484.
Email: litaiwen@scu.edu.cn (Taiwen Li) and 812471898@qq.com (Yu Zhou)

SUPPLEMENTARY FIGURES

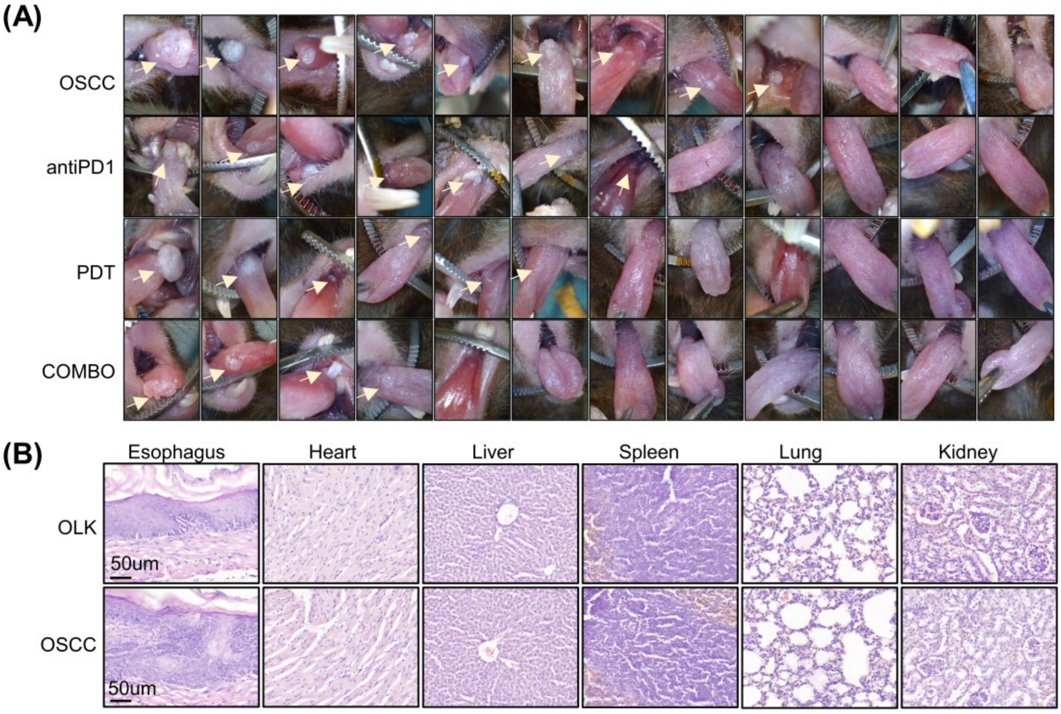

Figure S1. Additional details of the mice model.

(A) Images of tongue visible lesions.

(B) Representative images of H&E staining for esophagus, heart, liver, spleen, lungs, and kidney tissues of OLK and OSCC mice.

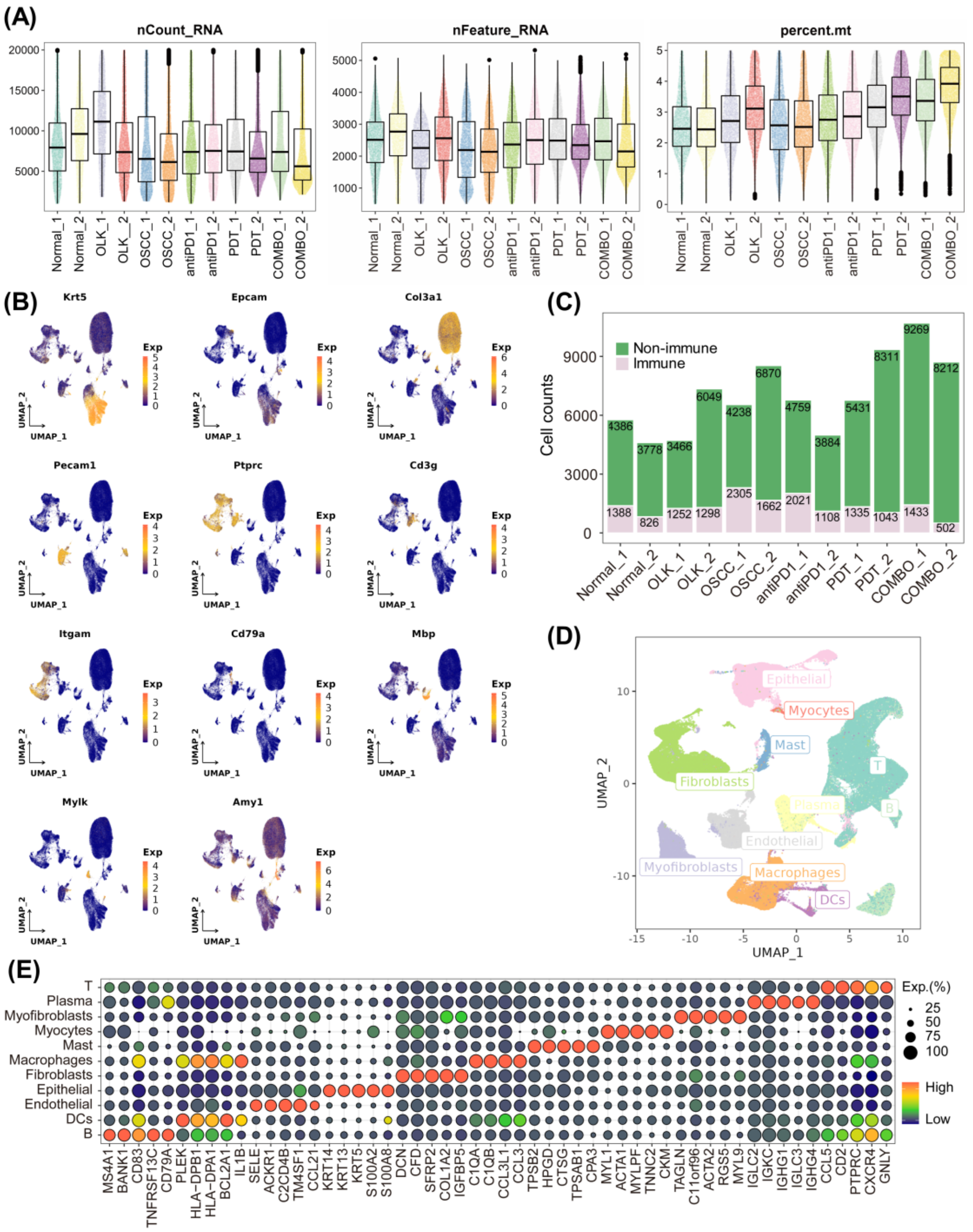

Figure S2. Additional details of the whole-cell atlas.

(A) Boxplot showing the number of unique molecular identifier (nCount_RNA) and genes (nFeature_RNA), and the frequency of mitochondrial ratio (percent.mt) in each sample. Each point in the graph represents an individual cell.

(B) UMAP of scale normalized expression of selected marker genes.

(C) Bar plot showing the number of immune cells and non-immune cells in each sample.

(D) UMAP plot visualizing celltypes of human scRNA-seq datasets (including two normal oral mucosal samples, three OLK samples, and three OSCC samples).

(E) Dotplot showing expression levels of marker genes for each celltype of human scRNA-seq datasets.

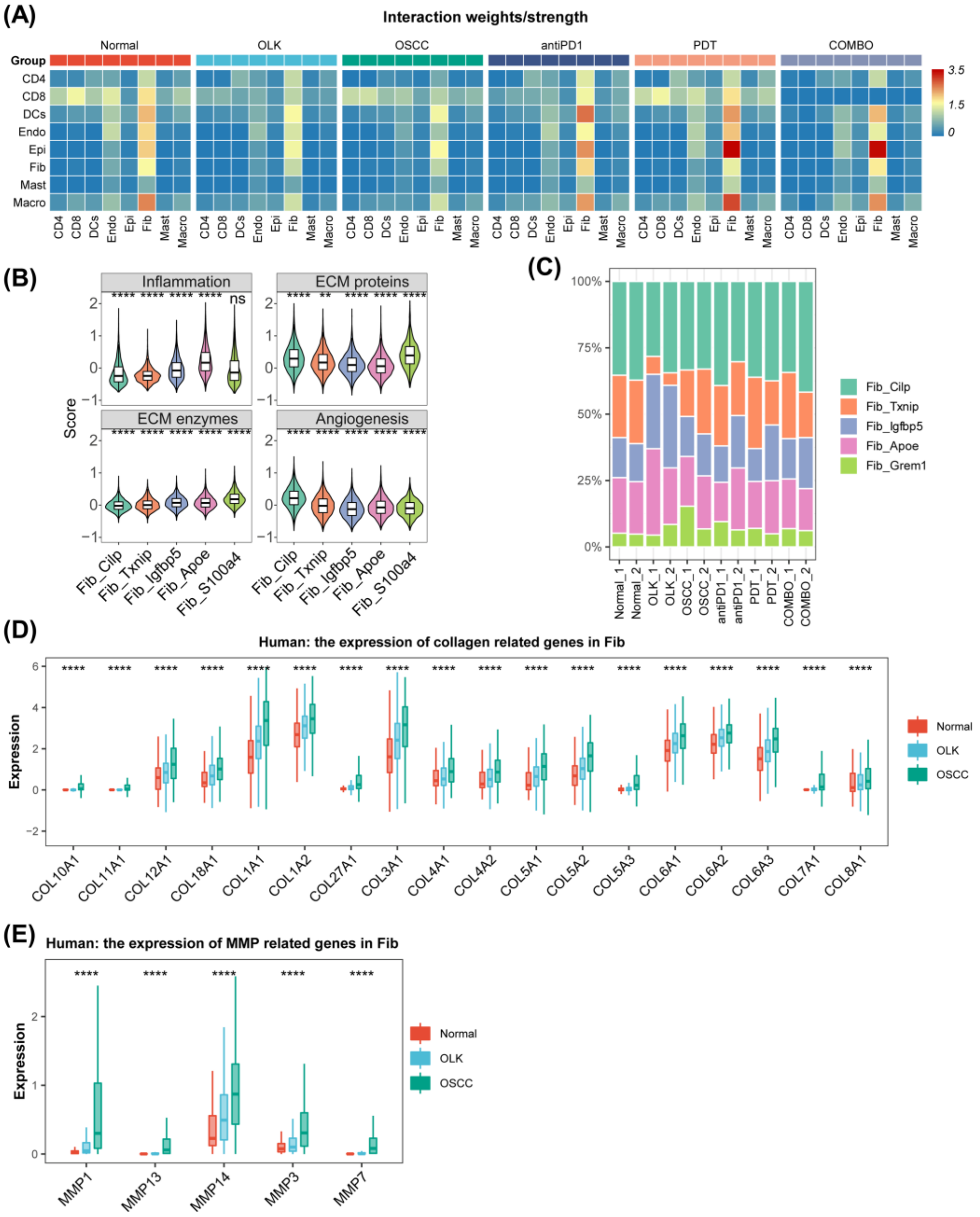

Figure S3. Additional details of fibroblasts.

(A) Heatmap showing the interaction weights/strength between celltypes across the six groups.

(B) Violin plot showing inflammation, ECM proteins, ECM enzymes, and angiogenesis related gene signature score of each group. ^{ns} P > 0.05, *P ≤ 0.05, **P ≤ 0.01, ***P ≤ 0.001, and ****P ≤ 0.0001 by Wilcoxon.

(C) Stacked bar plot showing the proportion of per fibroblast subtype in each sample.

(D) Box plot showing the expression level of collagen related genes in fibroblasts across groups in human scRNA-seq datasets. ^{ns} P > 0.05, *P ≤ 0.05, **P ≤ 0.01, ***P ≤ 0.001, and ****P ≤ 0.0001 by Wilcoxon.

(E) Box plot showing the expression level of matrix metalloproteinases (MMP) related genes in fibroblasts across groups in human scRNA-seq datasets. ^{ns} P > 0.05, *P ≤ 0.05, **P ≤ 0.01, ***P ≤ 0.001, and ****P ≤ 0.0001 by Wilcoxon.

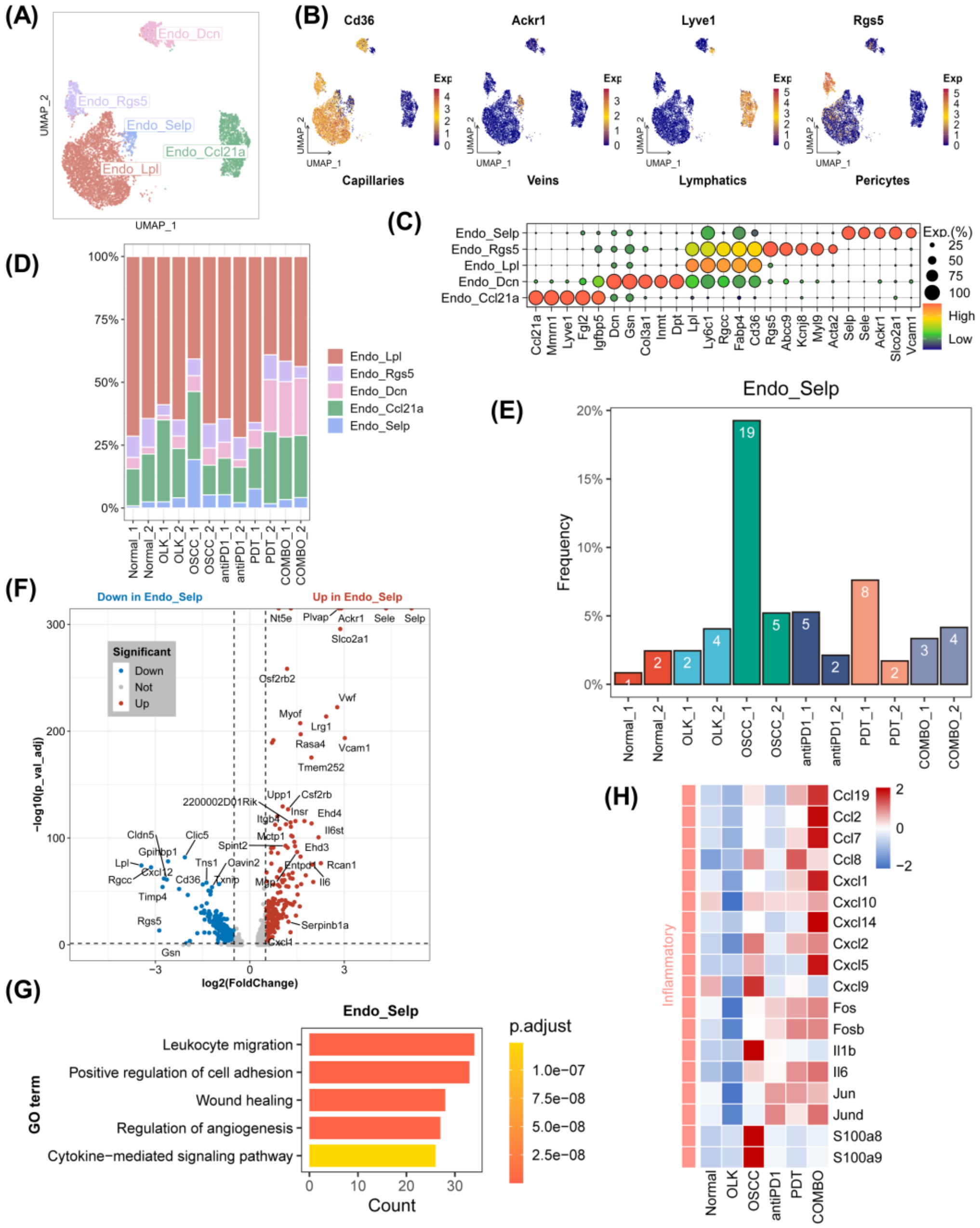

Figure S4. Endothelial subtypes and functional remodeling.

(A) UMAP plot visualizing the identified five endothelial subtypes (total = 6,482 cells).

(B) UMAP of scale normalized expression of selected marker genes.

(C) Dotplot showing expression levels of top-expression genes for each endothelial subtype.

(D) Stacked bar plot showing the proportion of per endothelial subtype in each sample.

(E) Bar plot showing the proportion of Endo_Selp in each sample.

(F) Volcano plot showing differentially expressed genes (DEGs) between Endo_Selp and other endothelial subtypes.

(G) Bar plot showing the selected GO biological processes with the upregulated DEGs in Endo_Selp. Adjusted *P*-value by Benjamini–Hochberg.

(H) Heatmap showing expression levels of inflammatory related genes across the six groups.

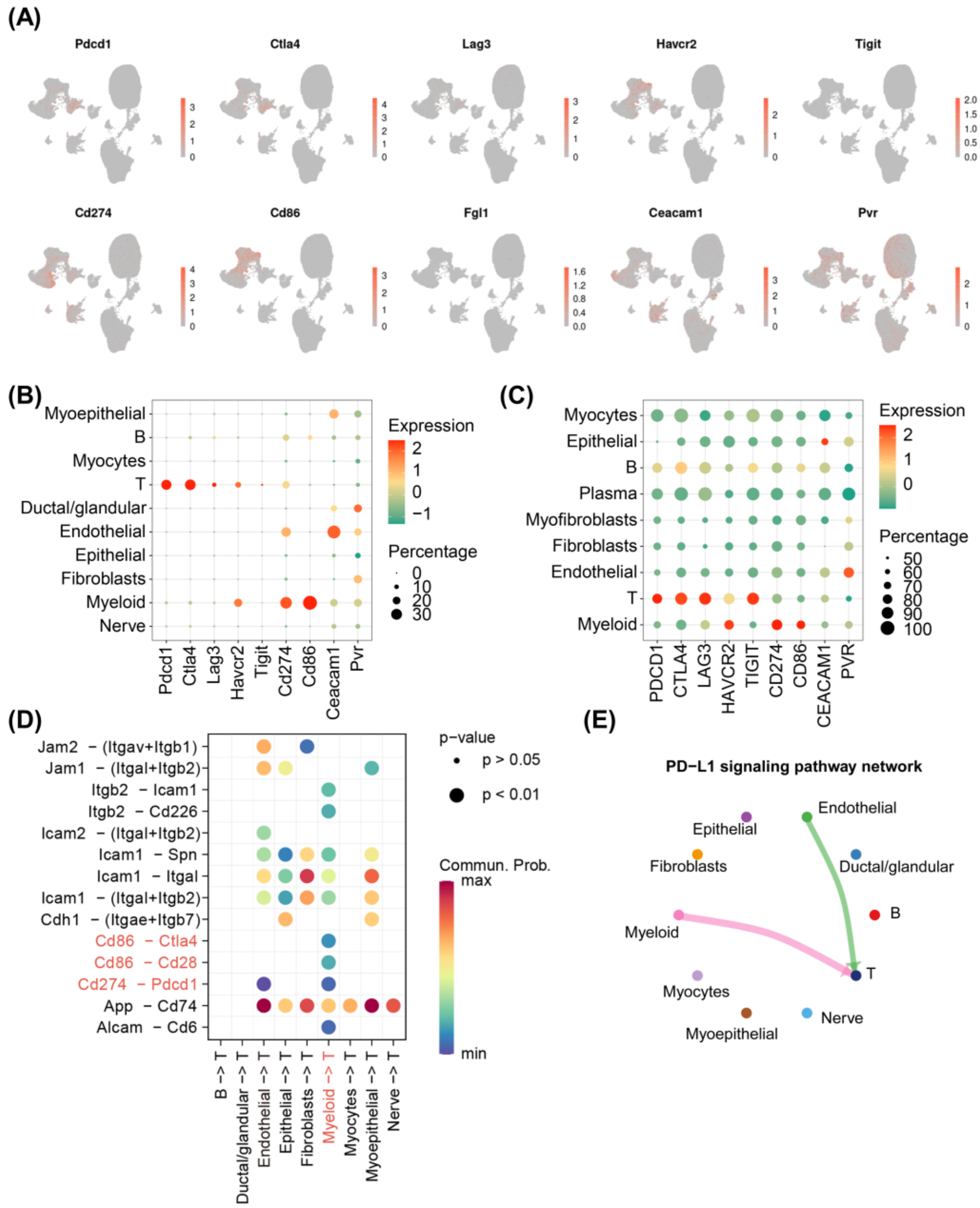

Figure S5. Myeloid cells are an important source which can inhibited the function of T cells.

(A) UMAP of scale normalized expression of immune checkpoints and their ligands in total cells.

(B) Scatter plot of the expression level of immune checkpoints and their ligands in each celltypes.

(C) Scatter plot of the expression level of immune checkpoints and their ligands in each celltypes in human scRNA-seq datasets.

(D) All the significant ligand-receptor pairs that contribute to the signaling sending from other celltypes to T cells. The dot color and size represent the calculated communication probability and *P*-values. *P*-values are computed from one-sided permutation test.

(E) The inferred PD-L1 signaling network. The edge width represents the communication probability.

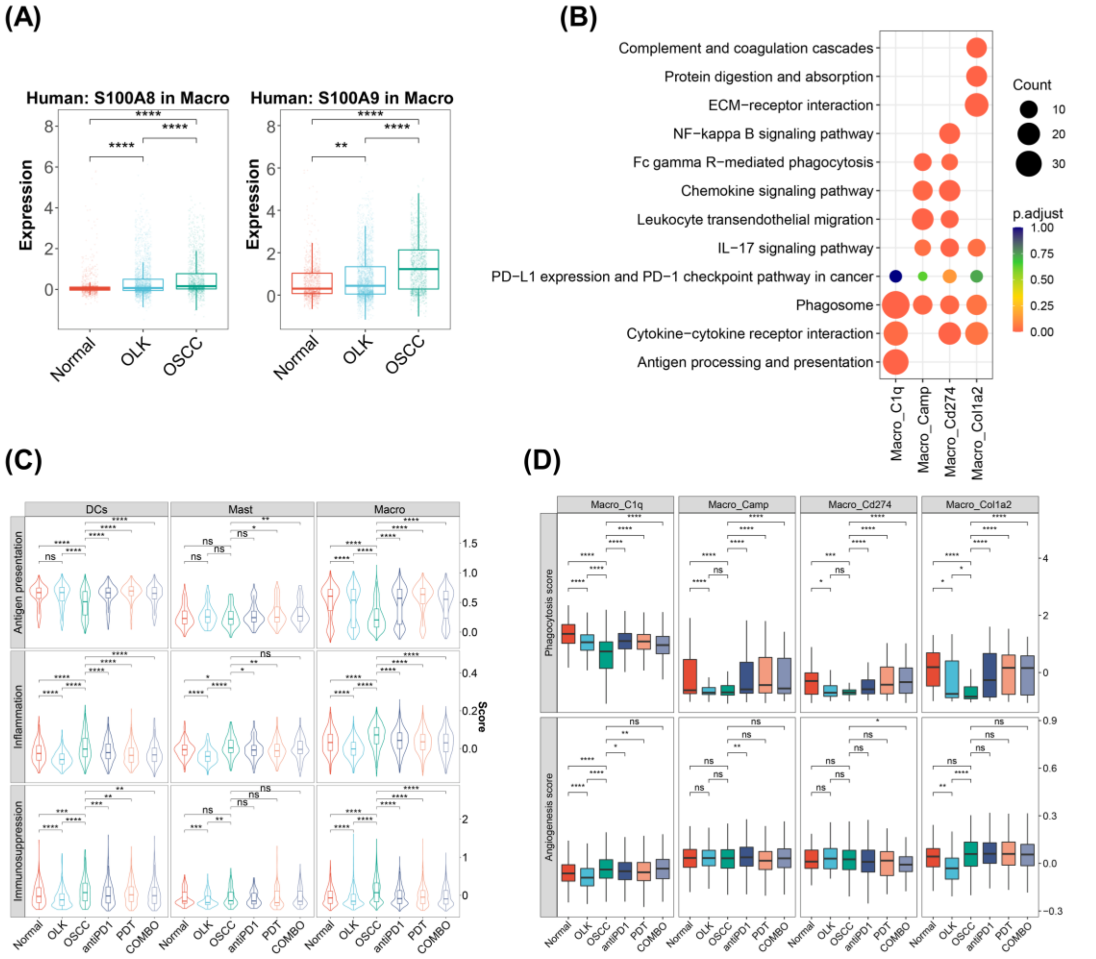

Figure S6. Additional details of myeloid cells.

(A) Box plot with jitter showing the expression level of *S100A8* (left) and *S100A9* (right) in total macrophages in human scRNA-seq datasets. $^{ns}P > 0.05$, $^{*}P \leq 0.05$, $^{**}P \leq 0.01$, $^{***}P \leq 0.001$, and $^{****}P \leq 0.0001$ by Wilcoxon.

(B) Scatter plot showing the selected KEGG terms enriched in macrophages' subtypes. Adjusted *P*-value by Benjamini–Hochberg.

(C) Violin plots showing antigen presentation, inflammation, and immunosuppression related gene signature score of each group in DCs, macrophages, and mast cells. $^{ns}P > 0.05$, $^{*}P \leq 0.05$, $^{**}P \leq 0.01$, $^{***}P \leq 0.001$, and $^{****}P \leq 0.0001$ by Wilcoxon.

(D) Box plots showing angiogenesis and phagocytosis related gene signature score of each group in macrophages' subtypes. $^{ns}P > 0.05$, $^{*}P \leq 0.05$, $^{**}P \leq 0.01$, $^{***}P \leq 0.001$, and $^{****}P \leq 0.0001$ by Wilcoxon.

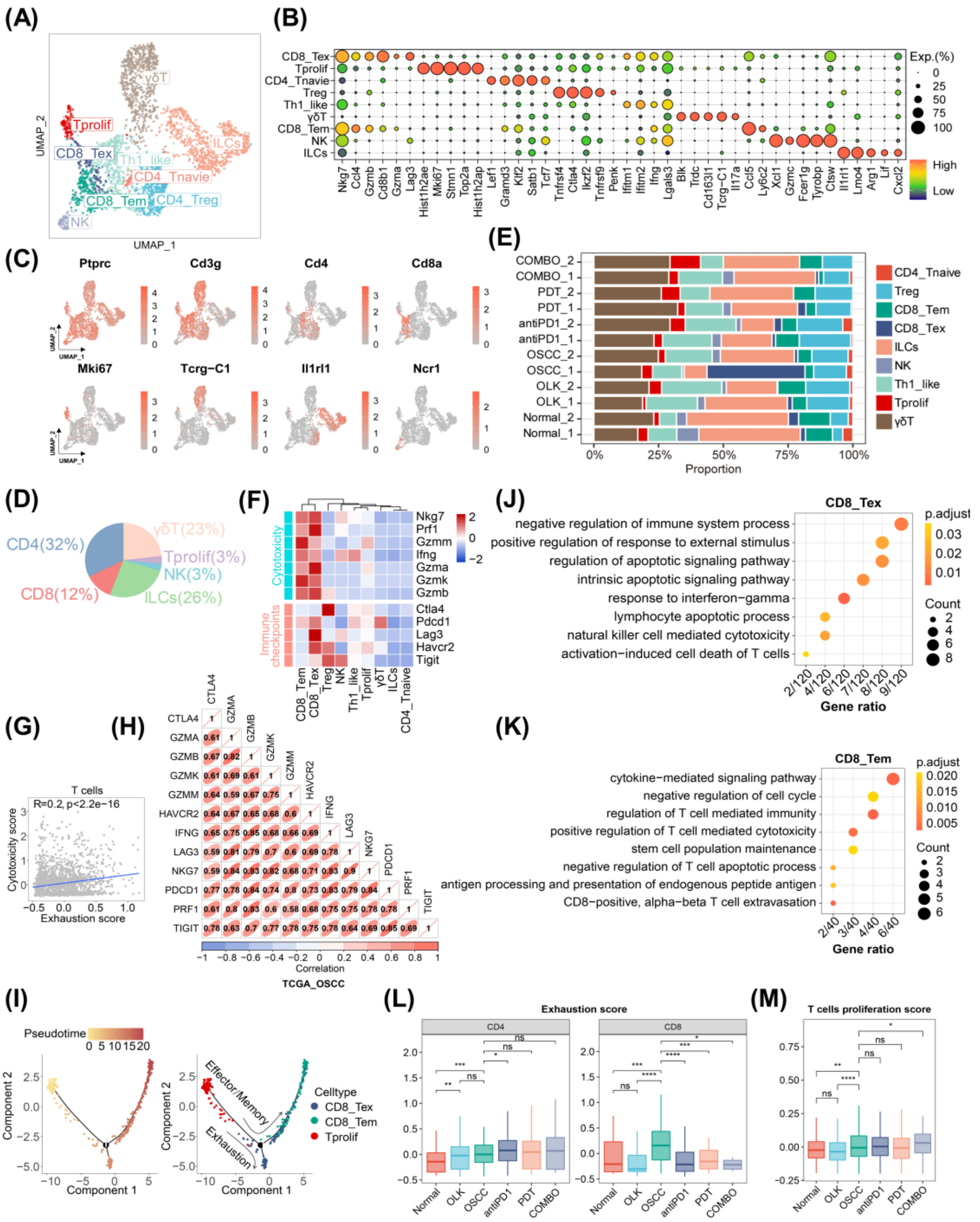

Figure S7. T cells subtypes and functional remodeling.

(A) UMAP plot visualizing the identified nine T cells' subtypes (n=2,840).

(B) Dotplot showing expression levels of top-expression genes for each T cells' subtype.

(C) UMAP of scale normalized expression of selected marker genes.

(D) Pie chart showing the proportion of each subset in total T cells.

(E) Box plot showing the proportion of per T cells' subtype in each sample.

(F) Heatmap showing expression levels of immune checkpoints and cytotoxicity related genes across the subtypes.

(G) The correlation between the exhaustion and cytotoxicity score in T cells by Pearson.

(H) The correlation between the expression level of immune checkpoints and

cytotoxicity related genes in TCGA OSCC cohorts.

(I) Transcriptional trajectory of $CD8^+$T cells inferred by Monocle2. Left: Points colored by pseudotime. Right: Points colored by cell subtypes.

(J) Scatter plot showing the selected GO biological processes with the upregulated DEGs in CD8_Tex. Adjusted *P*-value by Benjamini–Hochberg.

(K) Scatter plot showing the selected GO biological processes with the upregulated DEGs in CD8_Tem. Adjusted *P*-value by Benjamini–Hochberg.

(L) Box plots showing T cells exhaustion related gene signature score of each group. $^{ns}P > 0.05$, $*P \le 0.05$, $**P \le 0.01$, $***P \le 0.001$, and $****P \le 0.0001$ by Wilcoxon.

(M) Box plots showing T cells proliferation related gene signature score of each group. $^{ns}P > 0.05$, $*P \le 0.05$, $**P \le 0.01$, $***P \le 0.001$, and $****P \le 0.0001$ by Wilcoxon.

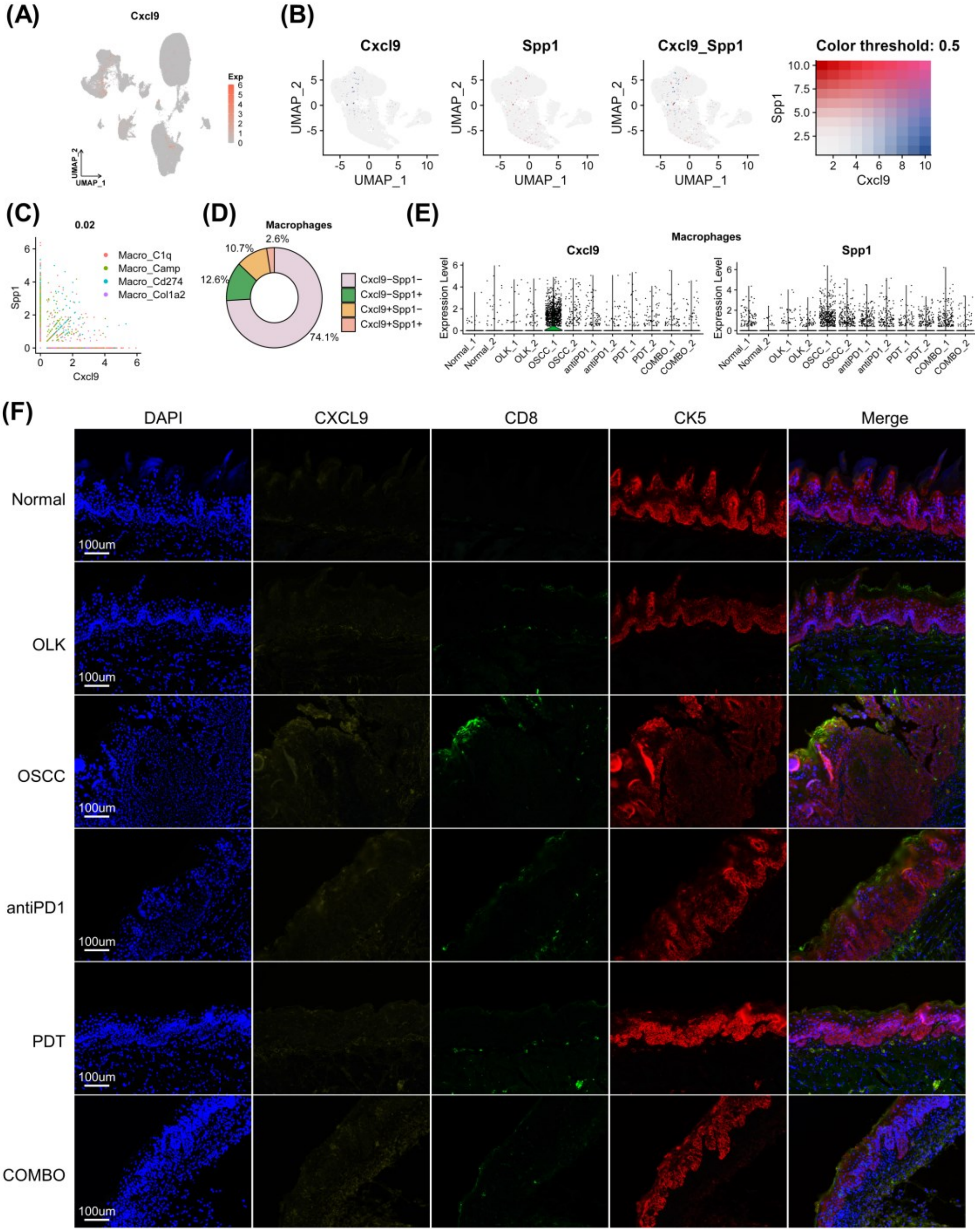

Figure S8. T cells subtypes and functional remodeling.

(A) UMAP plot visualizing the expression of *Cxcl9* in the whole-cell atlas.

(B) UMAP plot visualizing the expression of Cxcl9 in the myeloid cells.

(C) Scatter plot of *Cxcl9* and *Spp1*expression in macrophages.

(D) Pie chart showing the proportion of *Cxcl9*$^{+}$*SPP1*$^{-}$ macrophages, *Cxcl9*$^{+}$*SPP1*$^{+}$ macrophages, *Cxcl9*$^{-}$*SPP1*$^{-}$ macrophages, and *Cxcl9*$^{-}$*SPP1*$^{+}$ macrophages in total macrophages.

(E) Violin plot showing the expression of *Cxcl9* and *Spp1* in macrophages for each sample.

(F) mIHC staining of CXCL9, CD8, CK5 in mice tongue slides.

SUPPLEMENTARY TABLES

Table S1 The detailed information of classification for different cell types

Celltypes	Npcs	Dims	Resolution
Total	50	1:20	0.5
Epithelial cells	50	1:10	0.5
Fibroblasts	50	1:10	0.4
Endothelial cells	50	1:10	0.1
T cells	50	1:20	2
Myeloid cells	50	1:10	0.3

Table S2 The detailed information of the genesets used for calculating gene signature score

Function	Genesets
Cell_Proliferation	*Zwint, E2f1, Fen1, Foxm1, H2afz, Hmgb2, Mcm2, Mcm3, Mcm4, Mcm5, Mcm6, Mki67, Mybl2, Pcna, Plk1, Ccnd1, Aurka, Bub1, Top2a, Tyms, Dek, Ccnb1, Ccne149*
T_Cytotoxicity	*Gzma, Gzmb, Gzmm, Nkg7, Gnly, Prf1, Ifng*
T_Exhaustion	*Havcr2, Lag3, Tigit, Ctla4, Pdcd1, Layn*
Macro_Angiogenesis	*Ccnd2, Ccne1, Cd44, Cxcr4, E2f3, Edn1, Ezh2, Fgf18, Fgfr1, Fyn, Hey1, Itgav, Jag1, Jag2, Mmp9, Notch1, Pdgfa, Ptk2, Spp1, Stc1, Tnfaip6, Tymp, Vav2, Vcan, Vegfa*
Macro_Phagocytosis	*Mrc1, Cd163, Mertk, C1qb, C1qa, C1qc, Fcgr2b*
Myeloid_Immunosuppression	*Cd80, Cd86, Cd274, Pdcd1lg2*
Fib_Cytokines	*Ccl2, Ccl7, Ccl8, Ccl11, Ccl19, Cxcl1, Cxcl2, Cxcl12, Cxcl14, Cxcl10, Cxcl9, Cxcl5, Il6, Il17ra, Il33, Il1b*
ECM_proteins	*Col1a1, Col1a2, Col3a1, Col4a1, Col4a2, Col5a2, Col5a3, Col8a1, Col12a1, Col14a1, Col15a1, Col16a1, Col6a2, Col6a1, Col5a1, Col6a3*
ECM_enzymes	*Mmp2, Mmp3, Mmp14, Mmp19, Mmp23, Lox, Loxl2, Ctsc, Ctsk, Ctsh, Ctsd, Timp1, Timp2, Adamts1, Adamts2, Adamts4, Adamts5, Adamtsl4, Adam33*
Fib_Angiogenesis	*Pdgfrl, Pdgfra, Vegfc, Angptl1, Angptl2, Angptl7, Egfr*
